# Crystal structures of Ca_4+*x*_Y_3–*x*_Si_7_O_15+*x*_N_5–*x*_ (0 ≤ *x* ≤ 1) comprising of an isolated [Si_7_(O,N)_19_] unit

**DOI:** 10.1107/S2056989019001257

**Published:** 2019-01-25

**Authors:** Makoto Kobayashi, Takuya Yasunaga, Hideki Kato, Kotaro Fujii, Masatomo Yashima, Masato Kakihana

**Affiliations:** a Institute of Multidisciplinary Research for Advanced Materials, Tohoku University, 2-1-1 Katahira, Aoba-ku, Sendai 980-8577, Japan; b Department of Chemistry, School of Science, Tokyo Institute of Technology, 2-12-1-W4-17, Ookayama, Meguro-ku, To-kyo 152-8551, Japan

**Keywords:** oxynitride, silicate, crystal structure

## Abstract

Single crystals of the solid solution series Ca_4+*x*_Y_3–*x*_Si_7_O_15+*x*_N_5–*x*_ were obtained by a solid-state reaction method using a flux for *x* = 0, 0.5 and 1, resulting in Ca_4_Y_3_Si_7_O_15_N_5_ (tetra­calcium triyttrium hepta­silicon oxynitride), Ca_4.5_Y_2.5_Si_7_O_15.5_N_4.5_ and Ca_5_Y_2_Si_7_O_16_N_4_ (penta­calcium diyttrium hepta­silicon oxynitride). Single-crystal X-ray analysis revealed that the three compounds are isotypic and belong to space-group type *P*6_3_/*m*. Ca^2+^ and Y^3+^ cations are distributed over two crystallographic sites (site symmetry .. and 1) in a disordered manner. The corresponding (Ca,Y)-centred polyhedra are connected by edge-sharing, resulting in the formation of a layer structure extending parallel to (001). Three [Si(O,N)]_4_ tetra­hedra (two with point group symmetry *m*.., one with 3.. and half-occupancy) are condensed into an isolated [Si_7_(O,N)_19_] unit, in which an [Si(O,N)]_4_ tetra­hedron is located at the center of a 12-membered oxynitride ring with composition [Si_6_O_15_N_3_]. The present compounds are the first to have such an [Si_7_(O,N)_19_] unit in their structures.

## Abstract 2

The solid solution series Ca_4+*x*_Y_3–*x*_Si_7_O_15+*x*_N_5–*x*_ with *x* = 0, 0.5 and 1, crystallizes isotypically with a [Si_7_(O,N)_19_] unit as a characteristic building unit.

## Chemical context   

Silicon oxynitrides (or oxynitridosilicates) containing an alkaline-earth or a rare-earth metal cation have been extensively studied due to their potential applications as phosphors for white-light-emitting diodes (Takeda *et al.*, 2018 ▸). Recently, the exploration range for new silicon oxynitrides has been expanded to compounds with alkaline-earth and rare-earth metal cations. In this regard, Lu_4_Ba_2_[Si_9_ON_16_]O, Y_4_Ba_2_[Si_9_ON_16_]O (Maak *et al.*, 2017 ▸), La_3_BaSi_5_N_9_O_2_ (Durach *et al.*, 2015 ▸), Ca_1.4_Ce_2.6_Si_12_O_4.4_N_16.6_ (Park *et al.*, 2013 ▸), Ca_1.46_La_2.54_Si_12_O_4.45_N_16.55_ (Park *et al.*, 2012 ▸) or BaYSi_2_O_5_N (Kobayashi *et al.*, 2017 ▸) were synthesized and their crystal structures determined. The corresponding oxide or nitride forms are unknown for these materials. At the same time, the introduction of multiple anions contributes to the formation of otherwise unattainable silicate units in single anion compounds. In addition to the compounds mentioned above, for example, Ce_4_[Si_4_O_4_N_6_]O has a hyperbolic layer structure, which is composed of an [SiO_3_N] unit connected by three cyclic [Si_3_O_3_N_6_] units through corner-sharing (Irran *et al.*, 2000 ▸).

While exploring new oxynitrides, we obtained SrSiO_2.64_N_0.24_ with a single-chain inosilicate structure, which has not been realized for Sr- or Sr-rich metasilicate oxides and nitrides (Kobayashi *et al.*, 2018 ▸). In the present work, the synthesis and structure determination of three silicon oxynitrides, denoted by the solid solution series Ca_4+*x*_Y_3-*x*_Si_7_O_15+*x*_N_5–*x*_, with compositions of Ca_4_Y_3_Si_7_O_15_N_5_ (**1**, *x* = 0), Ca_4.5_Y_2.5_Si_7_O_15.5_N_4.5_ (**2**, *x* = 0.5) and Ca_5_Y_2_Si_7_O_16_N_4_ (**3**, *x* = 1) are reported.

## Structural commentary   

**Figure 1:**
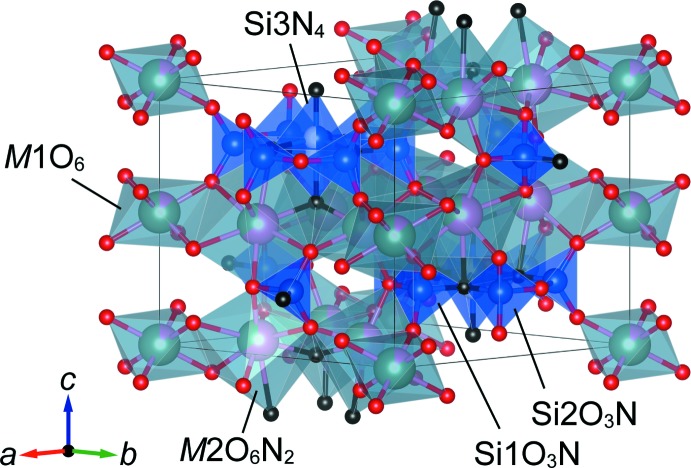
Crystal structure of Ca_4_Y_3_Si_7_O_15_N_5_ (**1**) drawn with cation-centered polyhedra. Pink, green, blue, red and black spheres indicate calcium, yttrium, silicon, oxygen, and nitro­gen ions, respectively. Mixed colours of the Si3 and associated (N,O) positions indicate the fraction of vacancy/occupancy.

Compounds **1**–**3** are isotypic and crystallize in space group *P*6_3_/*m*. Figs. 1 ▸–4 ▸ ▸ ▸ show the crystal structures and atomic arrangements of **1**–**3**. There are five sites in the structure associated with oxygen and/or nitro­gen positions. Two sites at Wyckoff position 12*i*, O1 and O2, and one 6*h* site, O3, are solely occupied by oxygen, and one 6*h* site, N5, is solely occupied by nitro­gen, irrespective of the value for *x*. Oxygen and nitro­gen atoms are disordered for (O,N)4 situated on a 4*f* site. For **2** and **3**, the O:N ratio at this site amounts to 0.25:0.75 for **2** and 0.5:0.5 for **3**, respectively, whereas for **1** this site is exclusively occupied by nitro­gen atoms.

**Figure 4:**
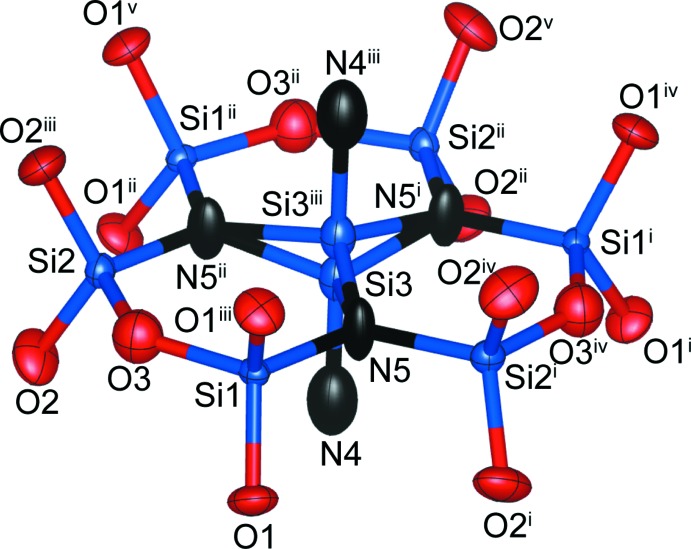
Representative for all structures, the atomic arrangement around Si atoms in the structure of Ca_4_Y_3_Si_7_O_15_N_5_ (**1**). Displacement ellipsoids are drawn at the 90% probability level. [Symmetry codes: (i) 1 − *y*, 1 + *x* − *y*, *z*; (ii) −*x* + *y*, 1 − *x*, *z*; (iii) *x*, *y*, 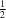 − *z*; (iv) 1 − *y*, 1 + *x* − *y*, 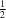 − *z*; (v) −*x* + *y*, 1 − *x*, 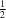 − *z*.]

Ca^2+^ and Y^3+^ occupy two sites, *viz. M*1 with site symmetry 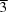.. at Wyckoff position 2*b* and *M*2 with site symmetry 1 at Wyckoff position 12*i. M*1 is coordinated by six oxygen atoms in the structures of **1**–**3** whereas the coordination environment of *M*2 depends on the value of *x*. In the structure of **1**, six oxygen and two nitro­gen atoms define the respective coord­in­ation sphere, but there are two possible coordination environments for **2** and **3** because of the disorder of the anionic 4*f* site, *i.e*. two nitro­gen and six oxygen atoms, and one nitro­gen and seven oxygen atoms, respectively. Site occupancies of Ca^2+^ were refined as 0.1379 (14) (**1**), 0.1338 (14) (**2**) and 0.2572 (14) (**3**) for *M*1, and 0.6437 (2) (**1**), 0.7277 (2) (**2**) and 0.7905 (2) (**3**) for *M*2. Thus, Ca^2+^ prefers to occupy the larger *M*2 site, in agreement with the larger ionic radius of Ca^2+^ compared to Y^3+^ for six- (1.00 *versus* 0.90 Å) and eight-coordination (1.12 *versus* 1.02 Å; Shannon, 1976 ▸). [*M*1O_6_] octa­hedra and [*M*2(O,N)_8_] dodeca­hedra are linked through their edges, resulting in the formation of a layer structure extending parallel to (001). Adjacent layers are connected along [001] through corner-sharing and by silicon atoms in the inter­stices. There are three Si sites in the crystal structure: Si1, Si2 and Si3 at Wyckhoff positions 6*h* (site symmetry *m*..), 6*h*, and 4*f* (3..), respectively. The latter site is disordered and shows half-occupancy. At both 6*h* sites, [SiO_3_N] tetra­hedra are present that are condensed into a 12-membered ring, [Si_6_O_15_N_3_], by corner-sharing. As a result of the disorder and associated splitting of the 4*f* site, tetra­hedra above and below the ring are present that share three corners with the ring, resulting in the formation of isolated [Si_7_(O,N)_19_] units, as shown in Fig. 4 ▸. These units are located at *z* = ±0.25 and are situated between the layers formed by layers of [*M*1O_6_] octa­hedra and [*M*2(O,N)_8_] dodeca­hedra.

Bond lengths of the [Si(O,N)_4_] tetra­hedra are collated in Table 1 ▸. In agreement with the higher electronegativity of oxygen when compared to nitro­gen, the Si—N bonds are systematically longer than Si—O bonds.

## Synthesis and crystallization   

Single crystals of **1**–**3** were obtained from powders synthesized by a solid-state reaction method. CaCO_3_ (Kanto Chemical, 99.99%), Y_2_O_3_ (Wako Chemical, 99.99%), SiO_2_ (Fuso Chemical, 99.999%), Si_3_N_4_ (Kojundo Chemical, 99.9%), and CeO_2_ (Kanto Chemical, 99.5%) in the molar ratio of CaCO_3_:Y_2_O_3_:SiO_2_:Si_3_N_4_ = 5.76:0.62:2.8:1.4 for **2** and of CaCO_3_:Y_2_O_3_:CeO_2_:SiO_2_:Si_3_N_4_ = 5.76:0.61:0.01:2.8:1.4 (2 mol% Ce to Y) for **1** and **3** were ground in the presence of 20 wt% CaF_2_ (Wako Chemical, 99.9%) as a flux. The mixtures were pelletized at 20 MPa, put on an alumina boat with a carbon sheet dish (Toyo Tanso, 0.1 mm of thickness) and calcined at 1733 K for 4 h under 100 ml min^−1^ of flowing nitro­gen. The reaction mixtures were slowly cooled under different conditions: to 1373 K at a rate of 30 K h^−1^, to 1173 K at a rate of 100 K h^−1^ and to RT by turning off the power for **1**, and to 1373 K at a rate of 60 K h^−1^, to 1173 K at a rate of 100 K h^−1^ and to RT by turning off the power for **2** and **3**. After roughly grinding the recrystallized fused pellets, the powders obtained were washed with 5 *M* HCl(aq.) and distilled water, followed by drying at 353 K. Colourless platelet-like single crystals were selected from the reaction products. Each crystal was cut into two portions. One was affixed to a Mitegen^(R)^ micro-mount device with a drop of Paratone N oil for single-crystal X-ray analysis. The other part was used for elemental analysis by energy dispersive X-ray (EDX) spectrometry using a scanning electron microscope (Hitachi, SU1510) equipped with an EDX detector (Horiba, X-act). EDX analysis indicated a Ca:Y:Si ratio of 0.266 (9):0.237 (4):0.497 (9) for **1**, of 0.325 (9):0.183 (5):0.492 (5) for **2**, and of 0.386 (19):0.146 (10):0.468 (10) for **3**. The ideal ratios according to the formulae of the three title compounds are 0.286:0.214:0.500, 0.321:0.179:0.500, and 0.357:0.143:0.500, respectively.

## Refinement   

Crystal data, data collection and structure refinement details are summarized in Table 2 ▸. In the initial stages of the refinements, all Si3 positions were treated as being located at the the 2*d* site, corresponding to a triangular environment of three N5 atoms. As a result of the strong anisotropy of the displacement ellipsoids along the *c* axis, the Si3 sites were subsequently refined as being split with half occupancy and a mirror symmetry element at *z* = ±0.25. When the occupancies of the disordered Ca^2+^ and Y^3+^ sites were refined freely, the ratios of Ca:Y were 7:86:6.14 for **1**, 10.07:3.93 for **2**, and 9:88:4.12 for **3**. Reliability factors for these refinements are summarized in Table 3 ▸. All values are almost the same, and the differences between the refined structures are within standard uncertainties. For the final steps of refinements, values obtained by EDX spectrometry were idealized under consideration of charge neutrality. Incorporation of Ce in single crystals of **1** and **3** was confirmed by their photoluminescence (the data are not shown). However, the contamination was ignored because of the small amount (2 mol% relative to Y, that is, Ca_4_Y_2.93_Ce_0.06_Si_7_O_15_N_5_ for **1** and Ca_5_Y_1.96_Ce_0.04_Si_7_O_15_N_5_ for **3**). Actually, consideration of the presence of Ce had only a marginal effect on refinement parameters and refined structures.

Five sites around the silicon atoms were detected. Although it is difficult to distinguish between oxygen and nitro­gen atoms by XRD analysis alone, site occupancies of oxygen and nitro­gen sites were determined from coordination environments, bond lengths, and bond-valence sums (Morgan, 1986 ▸; Fuertes, 2006 ▸; Braun *et al.*, 2010 ▸; Maak *et al.*, 2017 ▸). Following Pauling’s second crystal rule, the site at Wyckoff position 6*h* is coordinated by three Si atoms and thus should be occupied by nitro­gen (N5) alone. The relatively long bond length of Si3—(O,N)4, 1.804 (6), 1.769 (8), and 1.765 (7) Å for **1**, **2**, and **3**, respectively, indicate that the (O,N)4 site at the 4*f* position also might be occupied by nitro­gen. Under consideration of charge neutrality for the different compositions in **1**–**3**, this site was refined as being occupationally disordered by oxygen and nitro­gen for **2** and **3**.

## Supplementary Material

Crystal structure: contains datablock(s) global, 1, 2, 3, Publ_Block. DOI: 10.1107/S2056989019001257/wm5483sup1.cif

Structure factors: contains datablock(s) 1. DOI: 10.1107/S2056989019001257/wm54831sup2.hkl

Structure factors: contains datablock(s) 2. DOI: 10.1107/S2056989019001257/wm54832sup3.hkl

Structure factors: contains datablock(s) 3. DOI: 10.1107/S2056989019001257/wm54833sup4.hkl

CCDC references: 1892876, 1892875, 1892874

Additional supporting information:  crystallographic information; 3D view; checkCIF report

## Figures and Tables

**Figure 2:**
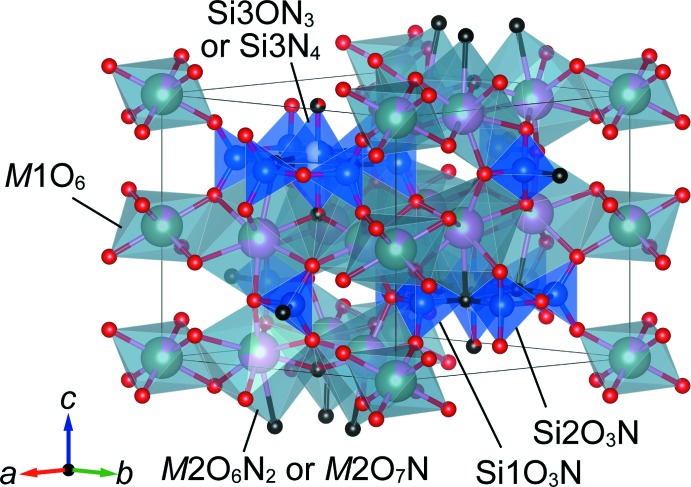
Crystal structure of Ca_4.5_Y_2.5_Si_7_O_15.5_N_4.5_ (**2**) drawn with cation-centered polyhedra. Colour code as in Fig. 1 ▸.

**Figure 3:**
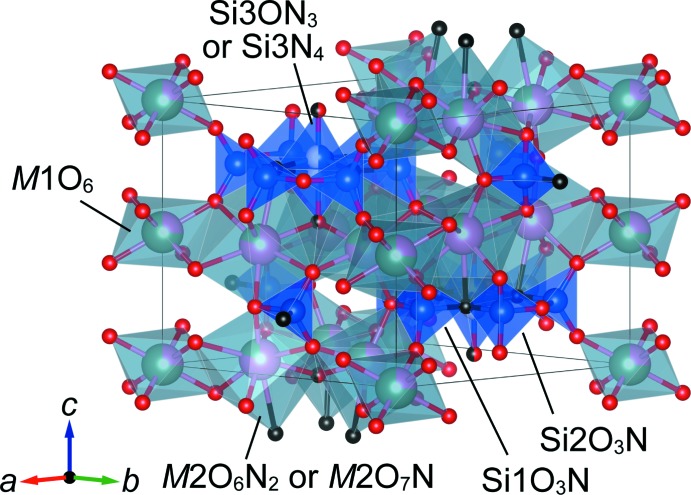
Crystal structure of Ca_5_Y_2_Si_7_O_16_N_4_ (**3**) drawn with cation-centered polyhedra. Colour code as in Fig. 1 ▸.

**Table 1:** Selected bond lengths (Å)

	**1**	**2**	**3**
Si1—O1	1.634 (2) × 2	1.621 (2) × 2	1.623 (2) × 2
Si1—O3	1.686 (3)	1.669 (4)	1.673 (3)
Si1—N5	1.754 (3)	1.788 (4)	1.781 (3)
Si2—O2	1.635 (2) × 2	1.622 (3) × 2	1.624 (2) × 2
Si2—O3	1.684 (3)	1.673 (4)	1.672 (3)
Si2—N5	1.753 (3)	1.798 (4)	1.782 (3)
Si3—(O,N)4	1.804 (6)	1.769 (8)	1.765 (7)
Si3—N5	1.809 (3) × 3	1.730 (5) × 3	1.732 (3) × 3

**Table 2:** Experimental details

	**1**	**2**	**3**
Crystal data			
Chemical formula	Ca_4_Y_3_Si_7_O_15_N_5_	Ca_4.5_Y_2.5_Si_7_O_15.5_N_4.5_	Ca_5_Y_2_Si_7_O_16_N_4_
*M*_r_	933.73	910.31	886.89
Crystal system, space group	Hexagonal, *P*6_3_/*m*	Hexagonal, *P*6_3_/*m*	Hexagonal, *P*6_3_/*m*
Temperature (K)	293	296	293
*a*, *c* (Å)	10.0884 (5), 9.9740 (5)	10.0792 (5), 9.9900 (5)	10.0541 (2), 10.0168 (2)
*V* (Å^3^)	879.11 (10)	878.92 (10)	876.89 (4)
*Z*	2	2	2
Radiation type	Mo *K*α	Mo *K*α	Mo *K*α
μ (mm^−1^)	11.56	10.08	8.63
Crystal size (mm)	0.07 × 0.04 × 0.01	0.07 × 0.06 × 0.01	0.05 × 0.03 × 0.02

Data collection			
Diffractometer	Rigaku XtaLAB PRO with a PILATUS 200K	Rigaku R-Axis RAPID II	Rigaku R-Axis RAPID II
Absorption correction	Multi-scan *(CrysAlis PRO*; Rigaku OD, 2015 ▸)	Multi-scan (*ABSCOR*; Higashi, 2001 ▸)	Multi-scan (*ABSCOR*; Higashi, 2001 ▸)
*T*_min_, *T*_max_	0.684, 1	0.724, 1.000	0.820, 1.000
No. of measured, independent and observed [*I* > 2σ(*I*)] reflections	8201, 778, 699	8568, 709, 688	8532, 702, 681
*R*_int_	0.039	0.030	0.027
(sin θ/λ)_max_ (Å^−1^)	0.685	0.649	0.648

Refinement			
*R*[*F* ^2^ > 2σ(*F* ^2^)], *wR*(*F* ^2^), *S*	0.025, 0.058, 1.10	0.033, 0.091, 1.19	0.024, 0.062, 1.09
No. of reflections	778	709	702
No. of parameters	62	63	63
No. of restraints	1	1	1
Δρ_max_, Δρ_min_ (e Å^−3^)	1.25, −0.56	1.83, −0.64	0.50, −0.86

Computer programs: *CrysAlis PRO* (Rigaku OD, 2015 ▸), *RAPID-AUTO* (Rigaku, 2005 ▸), *SHELXT* (Sheldrick, 2015*a* ▸), *SHELXL2014* (Sheldrick, 2015*b* ▸), *VESTA* (Momma & Izumi, 2011 ▸), *WinGX* (Farrugia, 2012 ▸) and *publCIF* (Westrip, 2010 ▸).

**Table 3:** *R*[*F* ^2^ > 2σ(*F* ^2^)], *wR*(*F* ^2^), *S* depending on the refinement of the site occupation factor of Ca and Y

	**1**	**2**	**3**
*R*[*F* ^2^ > 2*σ*(*F* ^2^)], *wR*(*F* ^2^), *S* at free occupancy	0.025, 0.058, 1.10	0.031, 0.088, 1.14	0.023, 0.062, 1.09
*R*[*F* ^2^ > 2*σ*(*F* ^2^)], *wR*(*F* ^2^), *S* at fixed occupancy	0.025, 0.058, 1.10	0.033, 0.091, 1.18	0.024, 0.062, 1.09

## supplementary crystallographic information

### Tetracalcium triyttrium heptasilicon oxynitride (1) Crystal data

**Table:**

Ca_4_Y_3_Si_7_O_15_N_5_	*D*_x_ = 3.527 Mg m^−^^3^
*M**_r_* = 933.73	Mo *K*α radiation, λ = 0.71073 Å
Hexagonal, *P*6_3_/*m*	Cell parameters from 2781 reflections
Hall symbol: -P 6c	θ = 3.0–28.8°
*a* = 10.0884 (5) Å	µ = 11.56 mm^−^^1^
*c* = 9.9740 (5) Å	*T* = 293 K
*V* = 879.11 (10) Å^3^	Platelet, colorless
*Z* = 2	0.07 × 0.04 × 0.01 mm
*F*(000) = 900	

### Tetracalcium triyttrium heptasilicon oxynitride (1) Data collection

**Table:**

Rigaku XtaLAB PRO with a PILATUS 200K diffractometer	778 independent reflections
Radiation source: fine-focus sealed X-ray tube	699 reflections with *I* > 2σ(*I*)
Graphite monochromator	*R*_int_ = 0.039
ω scans	θ_max_ = 29.1°, θ_min_ = 2.3°
Absorption correction: multi-scan (CrysAlisPro; Rigaku OD, 2015)	*h* = −13→13
*T*_min_ = 0.684, *T*_max_ = 1	*k* = −13→12
8201 measured reflections	*l* = −13→13

### Tetracalcium triyttrium heptasilicon oxynitride (1) Refinement

**Table:**

Refinement on *F*^2^	1 restraint
Least-squares matrix: full	0 constraints
*R*[*F*^2^ > 2σ(*F*^2^)] = 0.025	Primary atom site location: structure-invariant direct methods
*wR*(*F*^2^) = 0.058	*w* = 1/[σ^2^(*F*_o_^2^) + (0.0223*P*)^2^ + 2.2946*P*] where *P* = (*F*_o_^2^ + 2*F*_c_^2^)/3
*S* = 1.10	(Δ/σ)_max_ < 0.001
778 reflections	Δρ_max_ = 1.25 e Å^−^^3^
62 parameters	Δρ_min_ = −0.56 e Å^−^^3^

### Tetracalcium triyttrium heptasilicon oxynitride (1) Special details

**Table:**

Geometry. All esds (except the esd in the dihedral angle between two l.s. planes) are estimated using the full covariance matrix. The cell esds are taken into account individually in the estimation of esds in distances, angles and torsion angles; correlations between esds in cell parameters are only used when they are defined by crystal symmetry. An approximate (isotropic) treatment of cell esds is used for estimating esds involving l.s. planes.

### Tetracalcium triyttrium heptasilicon oxynitride (1) Fractional atomic coordinates and isotropic or equivalent isotropic displacement parameters (Å^2^)

**Table:**

	*x*	*y*	*z*	*U*_iso_*/*U*_eq_	Occ. (<1)
Ca1	0	0	0	0.00571 (16)	0.1379 (14)
Y1	0	0	0	0.00571 (16)	0.8620 (14)
Ca2	0.41947 (5)	0.13257 (4)	0.03127 (4)	0.01093 (14)	0.6437 (2)
Y2	0.41947 (5)	0.13257 (4)	0.03127 (4)	0.01093 (14)	0.3563 (2)
Si1	0.10867 (11)	0.31831 (11)	0.25	0.0059 (2)	
Si2	0.56028 (12)	0.01024 (11)	0.25	0.0075 (2)	
Si3	0.3333	0.6667	0.2191 (2)	0.0107 (8)	0.5
O1	0.0791 (2)	0.2237 (2)	0.10980 (19)	0.0136 (4)	
O2	0.4447 (2)	0.3571 (2)	0.1075 (2)	0.0170 (4)	
O3	0.3985 (3)	0.0201 (3)	0.25	0.0180 (6)	
N4	0.3333	0.6667	0.0382 (6)	0.0242 (11)	
N5	0.2961 (4)	0.4743 (4)	0.25	0.0164 (7)	

### Tetracalcium triyttrium heptasilicon oxynitride (1) Atomic displacement parameters (Å^2^)

**Table:**

	*U*^11^	*U*^22^	*U*^33^	*U*^12^	*U*^13^	*U*^23^
Ca1	0.0056 (2)	0.0056 (2)	0.0059 (3)	0.00282 (10)	0	0
Y1	0.0056 (2)	0.0056 (2)	0.0059 (3)	0.00282 (10)	0	0
Ca2	0.0164 (2)	0.0099 (2)	0.0095 (2)	0.00887 (17)	−0.00042 (15)	−0.00065 (14)
Y2	0.0164 (2)	0.0099 (2)	0.0095 (2)	0.00887 (17)	−0.00042 (15)	−0.00065 (14)
Si1	0.0059 (5)	0.0053 (5)	0.0056 (5)	0.0021 (4)	0	0
Si2	0.0061 (5)	0.0069 (5)	0.0087 (5)	0.0026 (4)	0	0
Si3	0.0083 (6)	0.0083 (6)	0.016 (2)	0.0041 (3)	0	0
O1	0.0166 (10)	0.0138 (9)	0.0100 (10)	0.0074 (8)	−0.0004 (8)	−0.0048 (8)
O2	0.0235 (11)	0.0159 (10)	0.0138 (10)	0.0114 (9)	−0.0054 (8)	−0.0069 (8)
O3	0.0171 (15)	0.0169 (15)	0.0204 (16)	0.0089 (12)	0	0
N4	0.0167 (14)	0.0167 (14)	0.039 (3)	0.0083 (7)	0	0
N5	0.0068 (16)	0.0096 (16)	0.032 (2)	0.0038 (13)	0	0

### Tetracalcium triyttrium heptasilicon oxynitride (1) Geometric parameters (Å, º)

**Table:**

Ca1—O1^i^	2.2646 (19)	Si2—Ca2^ix^	3.1707 (8)
Ca1—O1^ii^	2.2646 (19)	Si2—Ca2^viii^	3.2065 (6)
Ca1—O1^iii^	2.2646 (19)	Si2—Y2^viii^	3.2065 (6)
Ca1—O1^iv^	2.2646 (19)	Si2—Ca2^xiii^	3.2065 (6)
Ca1—O1^v^	2.2646 (19)	Si2—Y2^xiii^	3.2065 (6)
Ca1—O1	2.2646 (19)	Si3—Si3^ix^	0.617 (5)
Ca1—Y2^ii^	3.7596 (4)	Si3—N4	1.804 (6)
Ca1—Ca2^iii^	3.7596 (4)	Si3—N5^xiv^	1.809 (3)
Ca1—Ca2^ii^	3.7596 (4)	Si3—N5	1.809 (3)
Ca1—Y2^iii^	3.7596 (4)	Si3—N5^xv^	1.809 (4)
Ca1—Ca2^i^	3.7596 (4)	Si3—Ca2^xvi^	3.3919 (18)
Ca1—Y2^i^	3.7596 (4)	Si3—Y2^xvi^	3.3919 (18)
Ca2—O2	2.280 (2)	Si3—Ca2^ii^	3.3919 (18)
Ca2—N4^vi^	2.3979 (17)	Si3—Y2^ii^	3.3919 (18)
Ca2—O2^iv^	2.418 (2)	Si3—Ca2^vi^	3.3919 (18)
Ca2—O3	2.4189 (14)	Si3—Y2^vi^	3.3919 (18)
Ca2—O1^iv^	2.477 (2)	O1—Y2^ii^	2.477 (2)
Ca2—O2^vii^	2.624 (2)	O1—Ca2^ii^	2.477 (2)
Ca2—O1^iii^	2.634 (2)	O1—Ca2^v^	2.634 (2)
Ca2—N5^iv^	2.8519 (8)	O1—Y2^v^	2.634 (2)
Ca2—Si1^iii^	3.1641 (8)	O2—Si2^xvii^	1.635 (2)
Ca2—Si2	3.1707 (8)	O2—Y2^ii^	2.418 (2)
Ca2—Si2^viii^	3.2065 (6)	O2—Ca2^ii^	2.418 (2)
Ca2—Si1^iv^	3.2109 (6)	O2—Ca2^xvii^	2.624 (2)
Si1—O1	1.6342 (19)	O2—Y2^xvii^	2.624 (2)
Si1—O1^ix^	1.6342 (19)	O3—Si1^iii^	1.686 (3)
Si1—O3^v^	1.686 (3)	O3—Y2^ix^	2.4189 (14)
Si1—N5	1.754 (3)	O3—Ca2^ix^	2.4189 (14)
Si1—Ca2^v^	3.1641 (8)	N4—Y2^xvi^	2.3980 (17)
Si1—Y2^v^	3.1641 (8)	N4—Ca2^xvi^	2.3980 (17)
Si1—Ca2^x^	3.1641 (8)	N4—Ca2^vi^	2.3980 (17)
Si1—Y2^x^	3.1641 (8)	N4—Y2^vi^	2.3980 (17)
Si1—Ca2^xi^	3.2109 (6)	N4—Y2^ii^	2.3980 (17)
Si1—Y2^xi^	3.2109 (6)	N4—Ca2^ii^	2.3980 (17)
Si1—Ca2^ii^	3.2109 (6)	N5—Si2^xvii^	1.753 (3)
Si1—Y2^ii^	3.2109 (6)	N5—Si3^ix^	1.809 (3)
Si2—O2^xii^	1.635 (2)	N5—Ca2^ii^	2.8520 (8)
Si2—O2^vii^	1.635 (2)	N5—Y2^ii^	2.8520 (8)
Si2—O3	1.685 (3)	N5—Y2^xi^	2.8520 (8)
Si2—N5^vii^	1.753 (3)	N5—Ca2^xi^	2.8520 (8)
Si2—Y2^ix^	3.1707 (8)		
			
O1^i^—Ca1—O1^ii^	98.58 (6)	O2^xii^—Si2—O3	104.46 (9)
O1^i^—Ca1—O1^iii^	81.42 (6)	O2^vii^—Si2—O3	104.46 (9)
O1^ii^—Ca1—O1^iii^	180.00 (11)	O2^xii^—Si2—N5^vii^	107.17 (9)
O1^i^—Ca1—O1^iv^	98.58 (6)	O2^vii^—Si2—N5^vii^	107.17 (9)
O1^ii^—Ca1—O1^iv^	98.58 (6)	O3—Si2—N5^vii^	113.01 (16)
O1^iii^—Ca1—O1^iv^	81.42 (6)	O2^xii^—Si2—Y2^ix^	55.73 (8)
O1^i^—Ca1—O1^v^	81.42 (6)	O2^vii^—Si2—Y2^ix^	129.27 (9)
O1^ii^—Ca1—O1^v^	81.42 (6)	O3—Si2—Y2^ix^	48.77 (4)
O1^iii^—Ca1—O1^v^	98.58 (6)	N5^vii^—Si2—Y2^ix^	122.49 (7)
O1^iv^—Ca1—O1^v^	180.00 (9)	O2^xii^—Si2—Ca2^ix^	55.73 (8)
O1^i^—Ca1—O1	180	O2^vii^—Si2—Ca2^ix^	129.27 (9)
O1^ii^—Ca1—O1	81.42 (6)	O3—Si2—Ca2^ix^	48.77 (4)
O1^iii^—Ca1—O1	98.58 (6)	N5^vii^—Si2—Ca2^ix^	122.49 (7)
O1^iv^—Ca1—O1	81.42 (6)	Y2^ix^—Si2—Ca2^ix^	0
O1^v^—Ca1—O1	98.58 (6)	O2^xii^—Si2—Ca2	129.27 (9)
O1^i^—Ca1—Y2^ii^	140.44 (5)	O2^vii^—Si2—Ca2	55.73 (8)
O1^ii^—Ca1—Y2^ii^	80.35 (5)	O3—Si2—Ca2	48.77 (4)
O1^iii^—Ca1—Y2^ii^	99.65 (5)	N5^vii^—Si2—Ca2	122.49 (7)
O1^iv^—Ca1—Y2^ii^	43.64 (5)	Y2^ix^—Si2—Ca2	87
O1^v^—Ca1—Y2^ii^	136.36 (5)	Ca2^ix^—Si2—Ca2	86.95 (3)
O1—Ca1—Y2^ii^	39.56 (5)	O2^xii^—Si2—Ca2^viii^	147.48 (9)
O1^i^—Ca1—Ca2^iii^	39.56 (5)	O2^vii^—Si2—Ca2^viii^	47.33 (7)
O1^ii^—Ca1—Ca2^iii^	99.65 (5)	O3—Si2—Ca2^viii^	107.93 (5)
O1^iii^—Ca1—Ca2^iii^	80.35 (5)	N5^vii^—Si2—Ca2^viii^	62.33 (2)
O1^iv^—Ca1—Ca2^iii^	136.36 (5)	Y2^ix^—Si2—Ca2^viii^	156.7
O1^v^—Ca1—Ca2^iii^	43.64 (5)	Ca2^ix^—Si2—Ca2^viii^	156.70 (3)
O1—Ca1—Ca2^iii^	140.44 (5)	Ca2—Si2—Ca2^viii^	73.410 (12)
Y2^ii^—Ca1—Ca2^iii^	180	O2^xii^—Si2—Y2^viii^	147.48 (9)
O1^i^—Ca1—Ca2^ii^	140.44 (5)	O2^vii^—Si2—Y2^viii^	47.33 (7)
O1^ii^—Ca1—Ca2^ii^	80.35 (5)	O3—Si2—Y2^viii^	107.93 (5)
O1^iii^—Ca1—Ca2^ii^	99.65 (5)	N5^vii^—Si2—Y2^viii^	62.33 (2)
O1^iv^—Ca1—Ca2^ii^	43.64 (5)	Y2^ix^—Si2—Y2^viii^	156.70 (3)
O1^v^—Ca1—Ca2^ii^	136.36 (5)	Ca2^ix^—Si2—Y2^viii^	156.70 (3)
O1—Ca1—Ca2^ii^	39.56 (5)	Ca2—Si2—Y2^viii^	73.4
Y2^ii^—Ca1—Ca2^ii^	0	Ca2^viii^—Si2—Y2^viii^	0.000 (16)
Ca2^iii^—Ca1—Ca2^ii^	180.000 (13)	O2^xii^—Si2—Ca2^xiii^	47.33 (7)
O1^i^—Ca1—Y2^iii^	39.56 (5)	O2^vii^—Si2—Ca2^xiii^	147.48 (9)
O1^ii^—Ca1—Y2^iii^	99.65 (5)	O3—Si2—Ca2^xiii^	107.93 (5)
O1^iii^—Ca1—Y2^iii^	80.35 (5)	N5^vii^—Si2—Ca2^xiii^	62.33 (2)
O1^iv^—Ca1—Y2^iii^	136.36 (5)	Y2^ix^—Si2—Ca2^xiii^	73.4
O1^v^—Ca1—Y2^iii^	43.64 (5)	Ca2^ix^—Si2—Ca2^xiii^	73.410 (12)
O1—Ca1—Y2^iii^	140.44 (5)	Ca2—Si2—Ca2^xiii^	156.70 (3)
Y2^ii^—Ca1—Y2^iii^	180	Ca2^viii^—Si2—Ca2^xiii^	122.07 (3)
Ca2^iii^—Ca1—Y2^iii^	0.000 (16)	Y2^viii^—Si2—Ca2^xiii^	122.1
Ca2^ii^—Ca1—Y2^iii^	180	O2^xii^—Si2—Y2^xiii^	47.33 (7)
O1^i^—Ca1—Ca2^i^	80.35 (5)	O2^vii^—Si2—Y2^xiii^	147.48 (9)
O1^ii^—Ca1—Ca2^i^	43.64 (5)	O3—Si2—Y2^xiii^	107.93 (5)
O1^iii^—Ca1—Ca2^i^	136.36 (5)	N5^vii^—Si2—Y2^xiii^	62.33 (2)
O1^iv^—Ca1—Ca2^i^	140.44 (5)	Y2^ix^—Si2—Y2^xiii^	73.410 (12)
O1^v^—Ca1—Ca2^i^	39.56 (5)	Ca2^ix^—Si2—Y2^xiii^	73.410 (12)
O1—Ca1—Ca2^i^	99.65 (5)	Ca2—Si2—Y2^xiii^	156.7
Y2^ii^—Ca1—Ca2^i^	119.3	Ca2^viii^—Si2—Y2^xiii^	122.07 (3)
Ca2^iii^—Ca1—Ca2^i^	60.681 (2)	Y2^viii^—Si2—Y2^xiii^	122.07 (3)
Ca2^ii^—Ca1—Ca2^i^	119.319 (2)	Ca2^xiii^—Si2—Y2^xiii^	0.000 (16)
Y2^iii^—Ca1—Ca2^i^	60.7	Si3^ix^—Si3—N4	180
O1^i^—Ca1—Y2^i^	80.35 (5)	Si3^ix^—Si3—N5^xiv^	80.19 (7)
O1^ii^—Ca1—Y2^i^	43.64 (5)	N4—Si3—N5^xiv^	99.82 (7)
O1^iii^—Ca1—Y2^i^	136.36 (5)	Si3^ix^—Si3—N5	80.18 (7)
O1^iv^—Ca1—Y2^i^	140.44 (5)	N4—Si3—N5	99.82 (7)
O1^v^—Ca1—Y2^i^	39.56 (5)	N5^xiv^—Si3—N5	117.15 (4)
O1—Ca1—Y2^i^	99.65 (5)	Si3^ix^—Si3—N5^xv^	80.18 (8)
Y2^ii^—Ca1—Y2^i^	119.319 (2)	N4—Si3—N5^xv^	99.82 (7)
Ca2^iii^—Ca1—Y2^i^	60.681 (2)	N5^xiv^—Si3—N5^xv^	117.16 (4)
Ca2^ii^—Ca1—Y2^i^	119.319 (2)	N5—Si3—N5^xv^	117.15 (4)
Y2^iii^—Ca1—Y2^i^	60.681 (2)	Si3^ix^—Si3—Ca2^xvi^	137.41 (3)
Ca2^i^—Ca1—Y2^i^	0.000 (13)	N4—Si3—Ca2^xvi^	42.60 (3)
O2—Ca2—N4^vi^	72.84 (8)	N5^xiv^—Si3—Ca2^xvi^	57.22 (5)
O2—Ca2—O2^iv^	162.18 (6)	N5—Si3—Ca2^xvi^	117.10 (9)
N4^vi^—Ca2—O2^iv^	107.07 (10)	N5^xv^—Si3—Ca2^xvi^	117.54 (9)
O2—Ca2—O3	96.04 (8)	Si3^ix^—Si3—Y2^xvi^	137.41 (3)
N4^vi^—Ca2—O3	117.84 (14)	N4—Si3—Y2^xvi^	42.60 (3)
O2^iv^—Ca2—O3	99.44 (8)	N5^xiv^—Si3—Y2^xvi^	57.22 (5)
O2—Ca2—O1^iv^	80.58 (7)	N5—Si3—Y2^xvi^	117.10 (9)
N4^vi^—Ca2—O1^iv^	108.21 (10)	N5^xv^—Si3—Y2^xvi^	117.54 (9)
O2^iv^—Ca2—O1^iv^	82.66 (7)	Ca2^xvi^—Si3—Y2^xvi^	0.00 (2)
O3—Ca2—O1^iv^	130.56 (8)	Si3^ix^—Si3—Ca2^ii^	137.40 (3)
O2—Ca2—O2^vii^	113.34 (9)	N4—Si3—Ca2^ii^	42.60 (3)
N4^vi^—Ca2—O2^vii^	67.01 (7)	N5^xiv^—Si3—Ca2^ii^	117.54 (9)
O2^iv^—Ca2—O2^vii^	81.88 (7)	N5—Si3—Ca2^ii^	57.22 (5)
O3—Ca2—O2^vii^	62.54 (8)	N5^xv^—Si3—Ca2^ii^	117.10 (9)
O1^iv^—Ca2—O2^vii^	161.43 (7)	Ca2^xvi^—Si3—Ca2^ii^	71.77 (4)
O2—Ca2—O1^iii^	104.80 (7)	Y2^xvi^—Si3—Ca2^ii^	71.8
N4^vi^—Ca2—O1^iii^	177.55 (5)	Si3^ix^—Si3—Y2^ii^	137.40 (3)
O2^iv^—Ca2—O1^iii^	74.97 (6)	N4—Si3—Y2^ii^	42.60 (3)
O3—Ca2—O1^iii^	62.73 (8)	N5^xiv^—Si3—Y2^ii^	117.54 (9)
O1^iv^—Ca2—O1^iii^	70.53 (8)	N5—Si3—Y2^ii^	57.22 (5)
O2^vii^—Ca2—O1^iii^	114.87 (6)	N5^xv^—Si3—Y2^ii^	117.10 (9)
O2—Ca2—N5^iv^	104.57 (9)	Ca2^xvi^—Si3—Y2^ii^	71.77 (4)
N4^vi^—Ca2—N5^iv^	62.85 (14)	Y2^xvi^—Si3—Y2^ii^	71.77 (4)
O2^iv^—Ca2—N5^iv^	61.67 (8)	Ca2^ii^—Si3—Y2^ii^	0.000 (15)
O3—Ca2—N5^iv^	158.03 (9)	Si3^ix^—Si3—Ca2^vi^	137.40 (3)
O1^iv^—Ca2—N5^iv^	61.86 (8)	N4—Si3—Ca2^vi^	42.60 (3)
O2^vii^—Ca2—N5^iv^	101.48 (8)	N5^xiv^—Si3—Ca2^vi^	117.10 (9)
O1^iii^—Ca2—N5^iv^	117.64 (8)	N5—Si3—Ca2^vi^	117.54 (9)
O2—Ca2—Si1^iii^	103.50 (6)	N5^xv^—Si3—Ca2^vi^	57.22 (5)
N4^vi^—Ca2—Si1^iii^	149.56 (12)	Ca2^xvi^—Si3—Ca2^vi^	71.77 (4)
O2^iv^—Ca2—Si1^iii^	85.50 (5)	Y2^xvi^—Si3—Ca2^vi^	71.8
O3—Ca2—Si1^iii^	31.72 (7)	Ca2^ii^—Si3—Ca2^vi^	71.77 (4)
O1^iv^—Ca2—Si1^iii^	100.73 (5)	Y2^ii^—Si3—Ca2^vi^	71.8
O2^vii^—Ca2—Si1^iii^	88.24 (5)	Si3^ix^—Si3—Y2^vi^	137.40 (3)
O1^iii^—Ca2—Si1^iii^	31.06 (4)	N4—Si3—Y2^vi^	42.60 (3)
N5^iv^—Ca2—Si1^iii^	143.47 (7)	N5^xiv^—Si3—Y2^vi^	117.10 (9)
O2—Ca2—Si2	107.94 (6)	N5—Si3—Y2^vi^	117.54 (9)
N4^vi^—Ca2—Si2	92.78 (9)	N5^xv^—Si3—Y2^vi^	57.22 (5)
O2^iv^—Ca2—Si2	89.88 (5)	Ca2^xvi^—Si3—Y2^vi^	71.77 (4)
O3—Ca2—Si2	31.59 (7)	Y2^xvi^—Si3—Y2^vi^	71.77 (4)
O1^iv^—Ca2—Si2	158.96 (5)	Ca2^ii^—Si3—Y2^vi^	71.77 (4)
O2^vii^—Ca2—Si2	30.98 (5)	Y2^ii^—Si3—Y2^vi^	71.77 (4)
O1^iii^—Ca2—Si2	88.56 (5)	Ca2^vi^—Si3—Y2^vi^	0.000 (18)
N5^iv^—Ca2—Si2	130.63 (7)	Si1—O1—Ca1	150.03 (12)
Si1^iii^—Ca2—Si2	58.93 (3)	Si1—O1—Y2^ii^	100.71 (9)
O2—Ca2—Si2^viii^	137.54 (6)	Ca1—O1—Y2^ii^	104.82 (7)
N4^vi^—Ca2—Si2^viii^	81.59 (11)	Si1—O1—Ca2^ii^	100.71 (9)
O2^iv^—Ca2—Si2^viii^	29.80 (5)	Ca1—O1—Ca2^ii^	104.82 (7)
O3—Ca2—Si2^viii^	126.06 (7)	Y2^ii^—O1—Ca2^ii^	0
O1^iv^—Ca2—Si2^viii^	75.91 (5)	Si1—O1—Ca2^v^	92.67 (9)
O2^vii^—Ca2—Si2^viii^	85.57 (5)	Ca1—O1—Ca2^v^	99.97 (7)
O1^iii^—Ca2—Si2^viii^	100.00 (5)	Y2^ii^—O1—Ca2^v^	95.9
N5^iv^—Ca2—Si2^viii^	32.98 (7)	Ca2^ii^—O1—Ca2^v^	95.94 (7)
Si1^iii^—Ca2—Si2^viii^	115.22 (2)	Si1—O1—Y2^v^	92.67 (9)
Si2—Ca2—Si2^viii^	106.589 (12)	Ca1—O1—Y2^v^	99.97 (7)
O2—Ca2—Si1^iv^	86.68 (6)	Y2^ii^—O1—Y2^v^	95.94 (7)
N4^vi^—Ca2—Si1^iv^	82.26 (11)	Ca2^ii^—O1—Y2^v^	95.94 (7)
O2^iv^—Ca2—Si1^iv^	75.76 (5)	Ca2^v^—O1—Y2^v^	0.00 (3)
O3—Ca2—Si1^iv^	159.66 (7)	Si2^xvii^—O2—Ca2	139.08 (12)
O1^iv^—Ca2—Si1^iv^	30.01 (5)	Si2^xvii^—O2—Y2^ii^	102.87 (10)
O2^vii^—Ca2—Si1^iv^	134.39 (5)	Ca2—O2—Y2^ii^	107.8
O1^iii^—Ca2—Si1^iv^	97.06 (5)	Si2^xvii^—O2—Ca2^ii^	102.87 (10)
N5^iv^—Ca2—Si1^iv^	32.96 (7)	Ca2—O2—Ca2^ii^	107.85 (8)
Si1^iii^—Ca2—Si1^iv^	128.11 (3)	Y2^ii^—O2—Ca2^ii^	0
Si2—Ca2—Si1^iv^	162.54 (2)	Si2^xvii^—O2—Ca2^xvii^	93.30 (9)
Si2^viii^—Ca2—Si1^iv^	56.20 (2)	Ca2—O2—Ca2^xvii^	108.14 (8)
O1—Si1—O1^ix^	117.68 (15)	Y2^ii^—O2—Ca2^xvii^	98.1
O1—Si1—O3^v^	105.15 (9)	Ca2^ii^—O2—Ca2^xvii^	98.12 (7)
O1^ix^—Si1—O3^v^	105.15 (9)	Si2^xvii^—O2—Y2^xvii^	93.30 (9)
O1—Si1—N5	108.92 (9)	Ca2—O2—Y2^xvii^	108.1
O1^ix^—Si1—N5	108.92 (9)	Y2^ii^—O2—Y2^xvii^	98.12 (7)
O3^v^—Si1—N5	110.87 (16)	Ca2^ii^—O2—Y2^xvii^	98.12 (7)
O1—Si1—Ca2^v^	56.27 (7)	Ca2^xvii^—O2—Y2^xvii^	0.00 (3)
O1^ix^—Si1—Ca2^v^	128.66 (8)	Si2—O3—Si1^iii^	135.20 (19)
O3^v^—Si1—Ca2^v^	48.97 (4)	Si2—O3—Y2^ix^	99.64 (8)
N5—Si1—Ca2^v^	121.33 (7)	Si1^iii^—O3—Y2^ix^	99.31 (8)
O1—Si1—Y2^v^	56.27 (7)	Si2—O3—Ca2^ix^	99.64 (8)
O1^ix^—Si1—Y2^v^	128.66 (8)	Si1^iii^—O3—Ca2^ix^	99.31 (8)
O3^v^—Si1—Y2^v^	48.97 (4)	Y2^ix^—O3—Ca2^ix^	0
N5—Si1—Y2^v^	121.33 (7)	Si2—O3—Ca2	99.64 (8)
Ca2^v^—Si1—Y2^v^	0.000 (16)	Si1^iii^—O3—Ca2	99.31 (8)
O1—Si1—Ca2^x^	128.66 (8)	Y2^ix^—O3—Ca2	128.8
O1^ix^—Si1—Ca2^x^	56.27 (7)	Ca2^ix^—O3—Ca2	128.82 (13)
O3^v^—Si1—Ca2^x^	48.97 (4)	Si3—N4—Y2^xvi^	106.79 (13)
N5—Si1—Ca2^x^	121.33 (7)	Si3—N4—Ca2^xvi^	106.79 (13)
Ca2^v^—Si1—Ca2^x^	87.18 (3)	Y2^xvi^—N4—Ca2^xvi^	0
Y2^v^—Si1—Ca2^x^	87.2	Si3—N4—Ca2^vi^	106.79 (13)
O1—Si1—Y2^x^	128.66 (8)	Y2^xvi^—N4—Ca2^vi^	112
O1^ix^—Si1—Y2^x^	56.27 (7)	Ca2^xvi^—N4—Ca2^vi^	112.01 (12)
O3^v^—Si1—Y2^x^	48.97 (4)	Si3—N4—Y2^vi^	106.79 (13)
N5—Si1—Y2^x^	121.33 (7)	Y2^xvi^—N4—Y2^vi^	112.01 (12)
Ca2^v^—Si1—Y2^x^	87.18 (3)	Ca2^xvi^—N4—Y2^vi^	112.01 (12)
Y2^v^—Si1—Y2^x^	87.18 (3)	Ca2^vi^—N4—Y2^vi^	0.000 (19)
Ca2^x^—Si1—Y2^x^	0.000 (11)	Si3—N4—Y2^ii^	106.79 (13)
O1—Si1—Ca2^xi^	147.46 (9)	Y2^xvi^—N4—Y2^ii^	112.01 (12)
O1^ix^—Si1—Ca2^xi^	49.29 (7)	Ca2^xvi^—N4—Y2^ii^	112.01 (12)
O3^v^—Si1—Ca2^xi^	107.19 (5)	Ca2^vi^—N4—Y2^ii^	112.01 (12)
N5—Si1—Ca2^xi^	62.20 (2)	Y2^vi^—N4—Y2^ii^	112.01 (12)
Ca2^v^—Si1—Ca2^xi^	156.16 (3)	Si3—N4—Ca2^ii^	106.79 (13)
Y2^v^—Si1—Ca2^xi^	156.2	Y2^xvi^—N4—Ca2^ii^	112
Ca2^x^—Si1—Ca2^xi^	73.134 (8)	Ca2^xvi^—N4—Ca2^ii^	112.01 (12)
Y2^x^—Si1—Ca2^xi^	73.1	Ca2^vi^—N4—Ca2^ii^	112.01 (12)
O1—Si1—Y2^xi^	147.46 (9)	Y2^vi^—N4—Ca2^ii^	112
O1^ix^—Si1—Y2^xi^	49.29 (7)	Y2^ii^—N4—Ca2^ii^	0
O3^v^—Si1—Y2^xi^	107.19 (5)	Si2^xvii^—N5—Si1	119.1 (2)
N5—Si1—Y2^xi^	62.20 (2)	Si2^xvii^—N5—Si3^ix^	118.93 (18)
Ca2^v^—Si1—Y2^xi^	156.16 (3)	Si1—N5—Si3^ix^	121.00 (18)
Y2^v^—Si1—Y2^xi^	156.16 (3)	Si2^xvii^—N5—Si3	118.93 (18)
Ca2^x^—Si1—Y2^xi^	73.134 (8)	Si1—N5—Si3	121.00 (18)
Y2^x^—Si1—Y2^xi^	73.134 (8)	Si3^ix^—N5—Si3	19.64 (15)
Ca2^xi^—Si1—Y2^xi^	0.000 (18)	Si2^xvii^—N5—Ca2^ii^	84.69 (7)
O1—Si1—Ca2^ii^	49.29 (7)	Si1—N5—Ca2^ii^	84.84 (7)
O1^ix^—Si1—Ca2^ii^	147.46 (9)	Si3^ix^—N5—Ca2^ii^	110.18 (11)
O3^v^—Si1—Ca2^ii^	107.19 (5)	Si3—N5—Ca2^ii^	90.54 (9)
N5—Si1—Ca2^ii^	62.20 (2)	Si2^xvii^—N5—Y2^ii^	84.69 (7)
Ca2^v^—Si1—Ca2^ii^	73.134 (8)	Si1—N5—Y2^ii^	84.84 (7)
Y2^v^—Si1—Ca2^ii^	73.1	Si3^ix^—N5—Y2^ii^	110.18 (11)
Ca2^x^—Si1—Ca2^ii^	156.16 (3)	Si3—N5—Y2^ii^	90.54 (9)
Y2^x^—Si1—Ca2^ii^	156.2	Ca2^ii^—N5—Y2^ii^	0.00 (3)
Ca2^xi^—Si1—Ca2^ii^	121.79 (3)	Si2^xvii^—N5—Y2^xi^	84.69 (7)
Y2^xi^—Si1—Ca2^ii^	121.8	Si1—N5—Y2^xi^	84.84 (7)
O1—Si1—Y2^ii^	49.29 (7)	Si3^ix^—N5—Y2^xi^	90.54 (9)
O1^ix^—Si1—Y2^ii^	147.46 (9)	Si3—N5—Y2^xi^	110.18 (11)
O3^v^—Si1—Y2^ii^	107.19 (5)	Ca2^ii^—N5—Y2^xi^	159.27 (14)
N5—Si1—Y2^ii^	62.20 (2)	Y2^ii^—N5—Y2^xi^	159.27 (14)
Ca2^v^—Si1—Y2^ii^	73.134 (8)	Si2^xvii^—N5—Ca2^xi^	84.69 (7)
Y2^v^—Si1—Y2^ii^	73.134 (8)	Si1—N5—Ca2^xi^	84.84 (7)
Ca2^x^—Si1—Y2^ii^	156.16 (3)	Si3^ix^—N5—Ca2^xi^	90.54 (9)
Y2^x^—Si1—Y2^ii^	156.16 (3)	Si3—N5—Ca2^xi^	110.18 (11)
Ca2^xi^—Si1—Y2^ii^	121.79 (3)	Ca2^ii^—N5—Ca2^xi^	159.27 (14)
Y2^xi^—Si1—Y2^ii^	121.79 (3)	Y2^ii^—N5—Ca2^xi^	159.3
Ca2^ii^—Si1—Y2^ii^	0.000 (7)	Y2^xi^—N5—Ca2^xi^	0
O2^xii^—Si2—O2^vii^	120.73 (16)		

Symmetry codes: (i) −*x*, −*y*, −*z*; (ii) *x*−*y*, *x*, −*z*; (iii) −*x*+*y*, −*x*, *z*; (iv) *y*, −*x*+*y*, −*z*; (v) −*y*, *x*−*y*, *z*; (vi) −*x*+1, −*y*+1, −*z*; (vii) −*y*+1, *x*−*y*, *z*; (viii) −*x*+1, −*y*, −*z*; (ix) *x*, *y*, −*z*+1/2; (x) −*y*, *x*−*y*, −*z*+1/2; (xi) *x*−*y*, *x*, *z*+1/2; (xii) −*y*+1, *x*−*y*, −*z*+1/2; (xiii) −*x*+1, −*y*, *z*+1/2; (xiv) −*x*+*y*, −*x*+1, *z*; (xv) −*y*+1, *x*−*y*+1, *z*; (xvi) *y*, −*x*+*y*+1, −*z*; (xvii) −*x*+*y*+1, −*x*+1, *z*.

### Calcium yttrium heptasilicon oxynitride (2) Crystal data

**Table:**

Ca_4.5_Y_2.5_Si_7_O_15.5_N_4.5_	*D*_x_ = 3.44 Mg m^−^^3^
*M**_r_* = 910.31	Mo *K*α radiation, λ = 0.71073 Å
Hexagonal, *P*6_3_/*m*	Cell parameters from 6355 reflections
Hall symbol: -P 6c	θ = 3.1–27.5°
*a* = 10.0792 (5) Å	µ = 10.08 mm^−^^1^
*c* = 9.9900 (5) Å	*T* = 296 K
*V* = 878.92 (10) Å^3^	Platelet, colorless
*Z* = 2	0.07 × 0.06 × 0.01 mm
*F*(000) = 882	

### Calcium yttrium heptasilicon oxynitride (2) Data collection

**Table:**

Rigaku R-Axis RAPID II diffractometer	709 independent reflections
Radiation source: sealed x-ray tube	688 reflections with *I* > 2σ(*I*)
Graphite monochromator	*R*_int_ = 0.030
Detector resolution: 10 pixels mm^-1^	θ_max_ = 27.5°, θ_min_ = 3.1°
phi or ω oscillation scans	*h* = −13→12
Absorption correction: multi-scan (*ABSCOR*; Higashi, 2001)	*k* = −12→12
*T*_min_ = 0.724, *T*_max_ = 1.000	*l* = −12→12
8568 measured reflections	

### Calcium yttrium heptasilicon oxynitride (2) Refinement

**Table:**

Refinement on *F*^2^	0 constraints
Least-squares matrix: full	Primary atom site location: structure-invariant direct methods
*R*[*F*^2^ > 2σ(*F*^2^)] = 0.033	*w* = 1/[σ^2^(*F*_o_^2^) + (0.0508*P*)^2^ + 2.6144*P*] where *P* = (*F*_o_^2^ + 2*F*_c_^2^)/3
*wR*(*F*^2^) = 0.091	(Δ/σ)_max_ < 0.001
*S* = 1.19	Δρ_max_ = 1.83 e Å^−^^3^
709 reflections	Δρ_min_ = −0.64 e Å^−^^3^
63 parameters	Extinction correction: SHELXL-2014/7 (Sheldrick 2015b), Fc^*^=kFc[1+0.001xFc^2^λ^3^/sin(2θ)]^-1/4^
1 restraint	Extinction coefficient: 0.028 (2)

### Calcium yttrium heptasilicon oxynitride (2) Special details

**Table:**

Geometry. All esds (except the esd in the dihedral angle between two l.s. planes) are estimated using the full covariance matrix. The cell esds are taken into account individually in the estimation of esds in distances, angles and torsion angles; correlations between esds in cell parameters are only used when they are defined by crystal symmetry. An approximate (isotropic) treatment of cell esds is used for estimating esds involving l.s. planes.

### Calcium yttrium heptasilicon oxynitride (2) Fractional atomic coordinates and isotropic or equivalent isotropic displacement parameters (Å^2^)

**Table:**

	*x*	*y*	*z*	*U*_iso_*/*U*_eq_	Occ. (<1)
Ca1	0	0	0	0.0099 (3)	0.1338 (14)
Y1	0	0	0	0.0099 (3)	0.8662 (14)
Ca2	0.41517 (7)	0.12870 (6)	0.03382 (6)	0.0143 (2)	0.7277 (2)
Y2	0.41517 (7)	0.12870 (6)	0.03382 (6)	0.0143 (2)	0.2723 (2)
Si1	0.11057 (12)	0.31892 (13)	0.25	0.0061 (3)	
Si2	0.55909 (13)	0.01171 (14)	0.25	0.0077 (3)	
Si3	0.3333	0.6667	0.2216 (2)	0.0035 (9)	0.5
O1	0.0838 (3)	0.2250 (3)	0.1115 (2)	0.0143 (5)	
O2	0.4447 (3)	0.3570 (3)	0.1092 (3)	0.0184 (6)	
O3	0.3956 (4)	0.0167 (4)	0.25	0.0186 (8)	
O4	0.3333	0.6667	0.0445 (8)	0.0358 (15)	0.25
N4	0.3333	0.6667	0.0445 (8)	0.0358 (15)	0.75
N5	0.2989 (4)	0.4828 (5)	0.25	0.0251 (11)	

### Calcium yttrium heptasilicon oxynitride (2) Atomic displacement parameters (Å^2^)

**Table:**

	*U*^11^	*U*^22^	*U*^33^	*U*^12^	*U*^13^	*U*^23^
Ca1	0.0095 (3)	0.0095 (3)	0.0107 (4)	0.00475 (15)	0	0
Y1	0.0095 (3)	0.0095 (3)	0.0107 (4)	0.00475 (15)	0	0
Ca2	0.0209 (3)	0.0137 (3)	0.0117 (4)	0.0111 (2)	−0.0003 (2)	−0.00032 (19)
Y2	0.0209 (3)	0.0137 (3)	0.0117 (4)	0.0111 (2)	−0.0003 (2)	−0.00032 (19)
Si1	0.0049 (5)	0.0078 (6)	0.0045 (6)	0.0024 (4)	0	0
Si2	0.0060 (5)	0.0101 (6)	0.0072 (6)	0.0042 (4)	0	0
Si3	0.0015 (6)	0.0015 (6)	0.008 (3)	0.0007 (3)	0	0
O1	0.0198 (12)	0.0174 (11)	0.0076 (11)	0.0108 (10)	0.0010 (9)	−0.0036 (9)
O2	0.0295 (14)	0.0159 (11)	0.0107 (12)	0.0120 (10)	−0.0052 (10)	−0.0054 (10)
O3	0.0169 (17)	0.0204 (17)	0.0183 (19)	0.0092 (14)	0	0
O4	0.032 (2)	0.032 (2)	0.044 (4)	0.0159 (10)	0	0
N4	0.032 (2)	0.032 (2)	0.044 (4)	0.0159 (10)	0	0
N5	0.0081 (18)	0.020 (2)	0.044 (3)	0.0048 (16)	0	0

### Calcium yttrium heptasilicon oxynitride (2) Geometric parameters (Å, º)

**Table:**

Ca1—O1	2.276 (2)	Si2—Ca2^viii^	3.1415 (10)
Ca1—O1^i^	2.276 (2)	Si2—Y2^viii^	3.1415 (10)
Ca1—O1^ii^	2.276 (2)	Si2—Ca2^xii^	3.2368 (8)
Ca1—O1^iii^	2.276 (2)	Si2—Y2^xii^	3.2368 (8)
Ca1—O1^iv^	2.276 (2)	Si2—Y2^xiii^	3.2368 (8)
Ca1—O1^v^	2.276 (2)	Si2—Ca2^xiii^	3.2368 (8)
Ca1—Ca2	3.7255 (6)	Si3—Si3^viii^	0.567 (5)
Ca1—Ca2^iii^	3.7255 (6)	Si3—N5^xiv^	1.730 (5)
Ca1—Ca2^ii^	3.7255 (6)	Si3—N5^xv^	1.730 (5)
Ca1—Y2^ii^	3.7255 (6)	Si3—N5	1.730 (5)
Ca1—Y2^iii^	3.7255 (6)	Si3—O4	1.769 (8)
Ca2—O2	2.295 (2)	Si3—N4^viii^	2.336 (8)
Ca2—O3	2.3990 (17)	Si3—Ca2^xvi^	3.4587 (19)
Ca2—O2^iv^	2.422 (3)	Si3—Ca2^ii^	3.4587 (19)
Ca2—O1^iv^	2.457 (3)	Si3—Ca2^vi^	3.4587 (19)
Ca2—N4^vi^	2.463 (3)	O1—Y2^ii^	2.457 (3)
Ca2—O4^vi^	2.463 (3)	O1—Ca2^ii^	2.457 (3)
Ca2—O1^iii^	2.627 (2)	O1—Ca2^v^	2.627 (2)
Ca2—O2^vii^	2.638 (3)	O1—Y2^v^	2.627 (2)
Ca2—N5^iv^	2.9043 (12)	O2—Si2^xvii^	1.622 (3)
Ca2—Si1^iii^	3.1302 (10)	O2—Y2^ii^	2.422 (3)
Ca2—Si2	3.1415 (10)	O2—Ca2^ii^	2.422 (3)
Ca2—Si1^iv^	3.2255 (8)	O2—Ca2^xvii^	2.638 (3)
Si1—O1^viii^	1.621 (2)	O2—Y2^xvii^	2.638 (3)
Si1—O1	1.621 (2)	O3—Si1^iii^	1.669 (4)
Si1—O3^v^	1.669 (4)	O3—Ca2^viii^	2.3990 (17)
Si1—N5	1.788 (4)	O3—Y2^viii^	2.3990 (17)
Si1—Ca2^v^	3.1302 (10)	O4—Y2^xvi^	2.463 (3)
Si1—Y2^v^	3.1302 (10)	O4—Ca2^xvi^	2.463 (3)
Si1—Ca2^ix^	3.1302 (10)	O4—Ca2^vi^	2.463 (3)
Si1—Y2^ix^	3.1302 (10)	O4—Y2^vi^	2.463 (3)
Si1—Ca2^x^	3.2255 (8)	O4—Y2^ii^	2.463 (3)
Si1—Y2^x^	3.2255 (8)	O4—Ca2^ii^	2.463 (3)
Si1—Y2^ii^	3.2255 (8)	N5—Si3^viii^	1.730 (5)
Si1—Ca2^ii^	3.2255 (8)	N5—Si2^xvii^	1.798 (4)
Si2—O2^xi^	1.622 (3)	N5—Ca2^ii^	2.9042 (12)
Si2—O2^vii^	1.622 (3)	N5—Y2^ii^	2.9042 (12)
Si2—O3	1.673 (4)	N5—Y2^x^	2.9042 (12)
Si2—N5^vii^	1.798 (4)	N5—Ca2^x^	2.9042 (12)
			
O1—Ca1—O1^i^	180	Ca2^x^—Si1—Ca2^ii^	123.06 (4)
O1—Ca1—O1^ii^	81.90 (8)	Y2^x^—Si1—Ca2^ii^	123.1
O1^i^—Ca1—O1^ii^	98.10 (8)	Y2^ii^—Si1—Ca2^ii^	0
O1—Ca1—O1^iii^	98.10 (8)	O2^xi^—Si2—O2^vii^	120.21 (19)
O1^i^—Ca1—O1^iii^	81.90 (8)	O2^xi^—Si2—O3	105.94 (12)
O1^ii^—Ca1—O1^iii^	180.00 (14)	O2^vii^—Si2—O3	105.94 (12)
O1—Ca1—O1^iv^	81.90 (8)	O2^xi^—Si2—N5^vii^	107.38 (12)
O1^i^—Ca1—O1^iv^	98.10 (8)	O2^vii^—Si2—N5^vii^	107.38 (12)
O1^ii^—Ca1—O1^iv^	98.10 (8)	O3—Si2—N5^vii^	109.7 (2)
O1^iii^—Ca1—O1^iv^	81.90 (8)	O2^xi^—Si2—Ca2	130.42 (11)
O1—Ca1—O1^v^	98.10 (8)	O2^vii^—Si2—Ca2	57.07 (10)
O1^i^—Ca1—O1^v^	81.90 (8)	O3—Si2—Ca2	48.88 (6)
O1^ii^—Ca1—O1^v^	81.90 (8)	N5^vii^—Si2—Ca2	120.81 (9)
O1^iii^—Ca1—O1^v^	98.10 (8)	O2^xi^—Si2—Ca2^viii^	57.07 (10)
O1^iv^—Ca1—O1^v^	180.0 (2)	O2^vii^—Si2—Ca2^viii^	130.42 (11)
O1—Ca1—Ca2	79.39 (6)	O3—Si2—Ca2^viii^	48.88 (6)
O1^i^—Ca1—Ca2	100.61 (6)	N5^vii^—Si2—Ca2^viii^	120.81 (9)
O1^ii^—Ca1—Ca2	135.80 (6)	Ca2—Si2—Ca2^viii^	86.86 (4)
O1^iii^—Ca1—Ca2	44.20 (6)	O2^xi^—Si2—Y2^viii^	57.07 (10)
O1^iv^—Ca1—Ca2	39.84 (6)	O2^vii^—Si2—Y2^viii^	130.42 (11)
O1^v^—Ca1—Ca2	140.16 (6)	O3—Si2—Y2^viii^	48.88 (6)
O1—Ca1—Ca2^iii^	140.16 (6)	N5^vii^—Si2—Y2^viii^	120.81 (9)
O1^i^—Ca1—Ca2^iii^	39.84 (6)	Ca2—Si2—Y2^viii^	86.9
O1^ii^—Ca1—Ca2^iii^	100.61 (6)	Ca2^viii^—Si2—Y2^viii^	0.00 (2)
O1^iii^—Ca1—Ca2^iii^	79.39 (6)	O2^xi^—Si2—Ca2^xii^	145.99 (11)
O1^iv^—Ca1—Ca2^iii^	135.80 (6)	O2^vii^—Si2—Ca2^xii^	46.38 (9)
O1^v^—Ca1—Ca2^iii^	44.20 (6)	O3—Si2—Ca2^xii^	107.97 (6)
Ca2—Ca1—Ca2^iii^	119.187 (3)	N5^vii^—Si2—Ca2^xii^	63.05 (3)
O1—Ca1—Ca2^ii^	39.84 (6)	Ca2—Si2—Ca2^xii^	73.437 (16)
O1^i^—Ca1—Ca2^ii^	140.16 (6)	Ca2^viii^—Si2—Ca2^xii^	156.83 (4)
O1^ii^—Ca1—Ca2^ii^	79.39 (6)	Y2^viii^—Si2—Ca2^xii^	156.8
O1^iii^—Ca1—Ca2^ii^	100.61 (6)	O2^xi^—Si2—Y2^xii^	145.99 (11)
O1^iv^—Ca1—Ca2^ii^	44.20 (6)	O2^vii^—Si2—Y2^xii^	46.38 (9)
O1^v^—Ca1—Ca2^ii^	135.80 (6)	O3—Si2—Y2^xii^	107.97 (6)
Ca2—Ca1—Ca2^ii^	60.813 (3)	N5^vii^—Si2—Y2^xii^	63.05 (3)
Ca2^iii^—Ca1—Ca2^ii^	180.000 (18)	Ca2—Si2—Y2^xii^	73.4
O1—Ca1—Y2^ii^	39.84 (6)	Ca2^viii^—Si2—Y2^xii^	156.83 (4)
O1^i^—Ca1—Y2^ii^	140.16 (6)	Y2^viii^—Si2—Y2^xii^	156.83 (4)
O1^ii^—Ca1—Y2^ii^	79.39 (6)	Ca2^xii^—Si2—Y2^xii^	0.00 (4)
O1^iii^—Ca1—Y2^ii^	100.61 (6)	O2^xi^—Si2—Y2^xiii^	46.38 (9)
O1^iv^—Ca1—Y2^ii^	44.20 (6)	O2^vii^—Si2—Y2^xiii^	145.99 (11)
O1^v^—Ca1—Y2^ii^	135.80 (6)	O3—Si2—Y2^xiii^	107.97 (6)
Ca2—Ca1—Y2^ii^	60.8	N5^vii^—Si2—Y2^xiii^	63.05 (3)
Ca2^iii^—Ca1—Y2^ii^	180.000 (18)	Ca2—Si2—Y2^xiii^	156.8
Ca2^ii^—Ca1—Y2^ii^	0.00 (3)	Ca2^viii^—Si2—Y2^xiii^	73.437 (16)
O1—Ca1—Y2^iii^	140.16 (6)	Y2^viii^—Si2—Y2^xiii^	73.437 (16)
O1^i^—Ca1—Y2^iii^	39.84 (6)	Ca2^xii^—Si2—Y2^xiii^	122.32 (4)
O1^ii^—Ca1—Y2^iii^	100.61 (6)	Y2^xii^—Si2—Y2^xiii^	122.32 (4)
O1^iii^—Ca1—Y2^iii^	79.39 (6)	O2^xi^—Si2—Ca2^xiii^	46.38 (9)
O1^iv^—Ca1—Y2^iii^	135.80 (6)	O2^vii^—Si2—Ca2^xiii^	145.99 (11)
O1^v^—Ca1—Y2^iii^	44.20 (6)	O3—Si2—Ca2^xiii^	107.97 (6)
Ca2—Ca1—Y2^iii^	119.2	N5^vii^—Si2—Ca2^xiii^	63.05 (3)
Ca2^iii^—Ca1—Y2^iii^	0.00 (2)	Ca2—Si2—Ca2^xiii^	156.83 (4)
Ca2^ii^—Ca1—Y2^iii^	180.000 (14)	Ca2^viii^—Si2—Ca2^xiii^	73.437 (16)
Y2^ii^—Ca1—Y2^iii^	180.000 (14)	Y2^viii^—Si2—Ca2^xiii^	73.4
O2—Ca2—O3	96.63 (11)	Ca2^xii^—Si2—Ca2^xiii^	122.32 (4)
O2—Ca2—O2^iv^	161.52 (8)	Y2^xii^—Si2—Ca2^xiii^	122.3
O3—Ca2—O2^iv^	100.46 (10)	Y2^xiii^—Si2—Ca2^xiii^	0
O2—Ca2—O1^iv^	81.73 (8)	Si3^viii^—Si3—N5^xiv^	80.57 (8)
O3—Ca2—O1^iv^	132.32 (10)	Si3^viii^—Si3—N5^xv^	80.56 (9)
O2^iv^—Ca2—O1^iv^	81.59 (9)	N5^xiv^—Si3—N5^xv^	117.37 (5)
O2—Ca2—N4^vi^	72.63 (11)	Si3^viii^—Si3—N5	80.56 (8)
O3—Ca2—N4^vi^	119.23 (18)	N5^xiv^—Si3—N5	117.37 (5)
O2^iv^—Ca2—N4^vi^	104.53 (14)	N5^xv^—Si3—N5	117.36 (5)
O1^iv^—Ca2—N4^vi^	105.78 (14)	Si3^viii^—Si3—O4	180
O2—Ca2—O4^vi^	72.63 (11)	N5^xiv^—Si3—O4	99.44 (8)
O3—Ca2—O4^vi^	119.23 (18)	N5^xv^—Si3—O4	99.44 (8)
O2^iv^—Ca2—O4^vi^	104.53 (14)	N5—Si3—O4	99.44 (8)
O1^iv^—Ca2—O4^vi^	105.78 (14)	Si3^viii^—Si3—N4^viii^	0.0040 (10)
N4^vi^—Ca2—O4^vi^	0	N5^xiv^—Si3—N4^viii^	80.56 (8)
O2—Ca2—O1^iii^	106.08 (9)	N5^xv^—Si3—N4^viii^	80.56 (8)
O3—Ca2—O1^iii^	62.93 (10)	N5—Si3—N4^viii^	80.56 (8)
O2^iv^—Ca2—O1^iii^	75.98 (8)	O4—Si3—N4^viii^	180
O1^iv^—Ca2—O1^iii^	71.77 (10)	Si3^viii^—Si3—Ca2^xvi^	137.55 (3)
N4^vi^—Ca2—O1^iii^	177.46 (11)	N5^xiv^—Si3—Ca2^xvi^	56.98 (6)
O4^vi^—Ca2—O1^iii^	177.46 (11)	N5^xv^—Si3—Ca2^xvi^	117.04 (11)
O2—Ca2—O2^vii^	112.13 (12)	N5—Si3—Ca2^xvi^	116.96 (11)
O3—Ca2—O2^vii^	62.75 (10)	O4—Si3—Ca2^xvi^	42.46 (3)
O2^iv^—Ca2—O2^vii^	82.24 (9)	N4^viii^—Si3—Ca2^xvi^	137.54 (3)
O1^iv^—Ca2—O2^vii^	159.89 (8)	Si3^viii^—Si3—Ca2^ii^	137.54 (3)
N4^vi^—Ca2—O2^vii^	67.05 (10)	N5^xiv^—Si3—Ca2^ii^	117.04 (11)
O4^vi^—Ca2—O2^vii^	67.05 (10)	N5^xv^—Si3—Ca2^ii^	116.96 (11)
O1^iii^—Ca2—O2^vii^	115.49 (8)	N5—Si3—Ca2^ii^	56.98 (6)
O2—Ca2—N5^iv^	103.18 (11)	O4—Si3—Ca2^ii^	42.46 (3)
O3—Ca2—N5^iv^	157.70 (11)	N4^viii^—Si3—Ca2^ii^	137.54 (3)
O2^iv^—Ca2—N5^iv^	61.59 (11)	Ca2^xvi^—Si3—Ca2^ii^	71.55 (5)
O1^iv^—Ca2—N5^iv^	61.83 (10)	Si3^viii^—Si3—Ca2^vi^	137.54 (3)
N4^vi^—Ca2—N5^iv^	59.0 (2)	N5^xiv^—Si3—Ca2^vi^	116.96 (11)
O4^vi^—Ca2—N5^iv^	59.0 (2)	N5^xv^—Si3—Ca2^vi^	56.98 (6)
O1^iii^—Ca2—N5^iv^	119.67 (11)	N5—Si3—Ca2^vi^	117.04 (11)
O2^vii^—Ca2—N5^iv^	99.76 (10)	O4—Si3—Ca2^vi^	42.46 (3)
O2—Ca2—Si1^iii^	104.39 (7)	N4^viii^—Si3—Ca2^vi^	137.54 (3)
O3—Ca2—Si1^iii^	31.79 (9)	Ca2^xvi^—Si3—Ca2^vi^	71.55 (5)
O2^iv^—Ca2—Si1^iii^	86.87 (6)	Ca2^ii^—Si3—Ca2^vi^	71.55 (5)
O1^iv^—Ca2—Si1^iii^	102.18 (6)	Si1—O1—Ca1	150.45 (15)
N4^vi^—Ca2—Si1^iii^	151.01 (16)	Si1—O1—Y2^ii^	102.59 (12)
O4^vi^—Ca2—Si1^iii^	151.01 (16)	Ca1—O1—Y2^ii^	103.76 (9)
O1^iii^—Ca2—Si1^iii^	31.18 (5)	Si1—O1—Ca2^ii^	102.59 (12)
O2^vii^—Ca2—Si1^iii^	88.78 (6)	Ca1—O1—Ca2^ii^	103.76 (9)
N5^iv^—Ca2—Si1^iii^	145.42 (10)	Y2^ii^—O1—Ca2^ii^	0
O2—Ca2—Si2	107.34 (7)	Si1—O1—Ca2^v^	91.80 (10)
O3—Ca2—Si2	31.69 (8)	Ca1—O1—Ca2^v^	98.64 (9)
O2^iv^—Ca2—Si2	90.97 (7)	Y2^ii^—O1—Ca2^v^	95.7
O1^iv^—Ca2—Si2	160.68 (6)	Ca2^ii^—O1—Ca2^v^	95.69 (8)
N4^vi^—Ca2—Si2	93.31 (13)	Si1—O1—Y2^v^	91.80 (10)
O4^vi^—Ca2—Si2	93.31 (13)	Ca1—O1—Y2^v^	98.64 (9)
O1^iii^—Ca2—Si2	89.16 (6)	Y2^ii^—O1—Y2^v^	95.69 (8)
O2^vii^—Ca2—Si2	31.07 (6)	Ca2^ii^—O1—Y2^v^	95.69 (8)
N5^iv^—Ca2—Si2	129.41 (8)	Ca2^v^—O1—Y2^v^	0.00 (4)
Si1^iii^—Ca2—Si2	59.41 (3)	Si2^xvii^—O2—Ca2	139.04 (15)
O2—Ca2—Si1^iv^	86.75 (7)	Si2^xvii^—O2—Y2^ii^	104.62 (12)
O3—Ca2—Si1^iv^	160.80 (9)	Ca2—O2—Y2^ii^	106.1
O2^iv^—Ca2—Si1^iv^	74.80 (7)	Si2^xvii^—O2—Ca2^ii^	104.62 (12)
O1^iv^—Ca2—Si1^iv^	29.38 (6)	Ca2—O2—Ca2^ii^	106.14 (10)
N4^vi^—Ca2—Si1^iv^	79.85 (16)	Y2^ii^—O2—Ca2^ii^	0
O4^vi^—Ca2—Si1^iv^	79.85 (16)	Si2^xvii^—O2—Ca2^xvii^	91.86 (11)
O1^iii^—Ca2—Si1^iv^	97.94 (6)	Ca2—O2—Ca2^xvii^	109.92 (11)
O2^vii^—Ca2—Si1^iv^	133.13 (6)	Y2^ii^—O2—Ca2^xvii^	97.8
N5^iv^—Ca2—Si1^iv^	33.39 (8)	Ca2^ii^—O2—Ca2^xvii^	97.76 (9)
Si1^iii^—Ca2—Si1^iv^	129.12 (4)	Si2^xvii^—O2—Y2^xvii^	91.86 (11)
Si2—Ca2—Si1^iv^	161.90 (3)	Ca2—O2—Y2^xvii^	109.9
O1^viii^—Si1—O1	117.18 (18)	Y2^ii^—O2—Y2^xvii^	97.76 (9)
O1^viii^—Si1—O3^v^	106.15 (11)	Ca2^ii^—O2—Y2^xvii^	97.76 (9)
O1—Si1—O3^v^	106.15 (11)	Ca2^xvii^—O2—Y2^xvii^	0.00 (2)
O1^viii^—Si1—N5	109.23 (11)	Si1^iii^—O3—Si2	136.8 (2)
O1—Si1—N5	109.23 (11)	Si1^iii^—O3—Ca2	99.02 (10)
O3^v^—Si1—N5	108.6 (2)	Si2—O3—Ca2	99.42 (10)
O1^viii^—Si1—Ca2^v^	129.30 (10)	Si1^iii^—O3—Ca2^viii^	99.02 (10)
O1—Si1—Ca2^v^	57.02 (9)	Si2—O3—Ca2^viii^	99.42 (10)
O3^v^—Si1—Ca2^v^	49.19 (6)	Ca2—O3—Ca2^viii^	128.37 (16)
N5—Si1—Ca2^v^	120.21 (10)	Si1^iii^—O3—Y2^viii^	99.02 (10)
O1^viii^—Si1—Y2^v^	129.30 (10)	Si2—O3—Y2^viii^	99.42 (10)
O1—Si1—Y2^v^	57.02 (9)	Ca2—O3—Y2^viii^	128.4
O3^v^—Si1—Y2^v^	49.19 (6)	Ca2^viii^—O3—Y2^viii^	0.00 (3)
N5—Si1—Y2^v^	120.21 (10)	Si3—O4—Y2^xvi^	108.54 (18)
Ca2^v^—Si1—Y2^v^	0.00 (3)	Si3—O4—Ca2^xvi^	108.54 (18)
O1^viii^—Si1—Ca2^ix^	57.02 (9)	Y2^xvi^—O4—Ca2^xvi^	0
O1—Si1—Ca2^ix^	129.30 (10)	Si3—O4—Ca2^vi^	108.54 (18)
O3^v^—Si1—Ca2^ix^	49.19 (6)	Y2^xvi^—O4—Ca2^vi^	110.4
N5—Si1—Ca2^ix^	120.21 (10)	Ca2^xvi^—O4—Ca2^vi^	110.39 (17)
Ca2^v^—Si1—Ca2^ix^	87.25 (3)	Si3—O4—Y2^vi^	108.54 (18)
Y2^v^—Si1—Ca2^ix^	87.2	Y2^xvi^—O4—Y2^vi^	110.39 (17)
O1^viii^—Si1—Y2^ix^	57.02 (9)	Ca2^xvi^—O4—Y2^vi^	110.39 (17)
O1—Si1—Y2^ix^	129.30 (10)	Ca2^vi^—O4—Y2^vi^	0.00 (3)
O3^v^—Si1—Y2^ix^	49.19 (6)	Si3—O4—Y2^ii^	108.54 (18)
N5—Si1—Y2^ix^	120.21 (10)	Y2^xvi^—O4—Y2^ii^	110.39 (17)
Ca2^v^—Si1—Y2^ix^	87.25 (3)	Ca2^xvi^—O4—Y2^ii^	110.39 (17)
Y2^v^—Si1—Y2^ix^	87.25 (3)	Ca2^vi^—O4—Y2^ii^	110.39 (17)
Ca2^ix^—Si1—Y2^ix^	0.00 (3)	Y2^vi^—O4—Y2^ii^	110.39 (17)
O1^viii^—Si1—Ca2^x^	48.03 (9)	Si3—O4—Ca2^ii^	108.54 (18)
O1—Si1—Ca2^x^	146.28 (10)	Y2^xvi^—O4—Ca2^ii^	110.4
O3^v^—Si1—Ca2^x^	107.30 (6)	Ca2^xvi^—O4—Ca2^ii^	110.39 (17)
N5—Si1—Ca2^x^	63.39 (3)	Ca2^vi^—O4—Ca2^ii^	110.39 (17)
Ca2^v^—Si1—Ca2^x^	156.46 (4)	Y2^vi^—O4—Ca2^ii^	110.4
Y2^v^—Si1—Ca2^x^	156.5	Y2^ii^—O4—Ca2^ii^	0
Ca2^ix^—Si1—Ca2^x^	72.773 (9)	Si3^viii^—N5—Si3	18.88 (17)
Y2^ix^—Si1—Ca2^x^	72.8	Si3^viii^—N5—Si1	122.8 (2)
O1^viii^—Si1—Y2^x^	48.03 (9)	Si3—N5—Si1	122.8 (2)
O1—Si1—Y2^x^	146.28 (10)	Si3^viii^—N5—Si2^xvii^	121.1 (2)
O3^v^—Si1—Y2^x^	107.30 (6)	Si3—N5—Si2^xvii^	121.1 (2)
N5—Si1—Y2^x^	63.39 (3)	Si1—N5—Si2^xvii^	115.1 (3)
Ca2^v^—Si1—Y2^x^	156.46 (4)	Si3^viii^—N5—Ca2^ii^	111.94 (15)
Y2^v^—Si1—Y2^x^	156.46 (4)	Si3—N5—Ca2^ii^	93.06 (10)
Ca2^ix^—Si1—Y2^x^	72.773 (9)	Si1—N5—Ca2^ii^	83.21 (9)
Y2^ix^—Si1—Y2^x^	72.773 (9)	Si2^xvii^—N5—Ca2^ii^	83.45 (9)
Ca2^x^—Si1—Y2^x^	0.00 (3)	Si3^viii^—N5—Y2^ii^	111.94 (15)
O1^viii^—Si1—Y2^ii^	146.28 (10)	Si3—N5—Y2^ii^	93.06 (10)
O1—Si1—Y2^ii^	48.03 (9)	Si1—N5—Y2^ii^	83.21 (9)
O3^v^—Si1—Y2^ii^	107.30 (6)	Si2^xvii^—N5—Y2^ii^	83.45 (9)
N5—Si1—Y2^ii^	63.39 (3)	Ca2^ii^—N5—Y2^ii^	0.00 (4)
Ca2^v^—Si1—Y2^ii^	72.773 (9)	Si3^viii^—N5—Y2^x^	93.06 (10)
Y2^v^—Si1—Y2^ii^	72.773 (9)	Si3—N5—Y2^x^	111.94 (15)
Ca2^ix^—Si1—Y2^ii^	156.46 (4)	Si1—N5—Y2^x^	83.21 (9)
Y2^ix^—Si1—Y2^ii^	156.46 (4)	Si2^xvii^—N5—Y2^x^	83.45 (9)
Ca2^x^—Si1—Y2^ii^	123.06 (4)	Ca2^ii^—N5—Y2^x^	155.00 (19)
Y2^x^—Si1—Y2^ii^	123.06 (4)	Y2^ii^—N5—Y2^x^	155.00 (19)
O1^viii^—Si1—Ca2^ii^	146.28 (10)	Si3^viii^—N5—Ca2^x^	93.06 (10)
O1—Si1—Ca2^ii^	48.03 (9)	Si3—N5—Ca2^x^	111.94 (15)
O3^v^—Si1—Ca2^ii^	107.30 (6)	Si1—N5—Ca2^x^	83.21 (9)
N5—Si1—Ca2^ii^	63.39 (3)	Si2^xvii^—N5—Ca2^x^	83.45 (9)
Ca2^v^—Si1—Ca2^ii^	72.773 (9)	Ca2^ii^—N5—Ca2^x^	155.00 (19)
Y2^v^—Si1—Ca2^ii^	72.8	Y2^ii^—N5—Ca2^x^	155
Ca2^ix^—Si1—Ca2^ii^	156.46 (4)	Y2^x^—N5—Ca2^x^	0
Y2^ix^—Si1—Ca2^ii^	156.5		

Symmetry codes: (i) −*x*, −*y*, −*z*; (ii) *x*−*y*, *x*, −*z*; (iii) −*x*+*y*, −*x*, *z*; (iv) *y*, −*x*+*y*, −*z*; (v) −*y*, *x*−*y*, *z*; (vi) −*x*+1, −*y*+1, −*z*; (vii) −*y*+1, *x*−*y*, *z*; (viii) *x*, *y*, −*z*+1/2; (ix) −*y*, *x*−*y*, −*z*+1/2; (x) *x*−*y*, *x*, *z*+1/2; (xi) −*y*+1, *x*−*y*, −*z*+1/2; (xii) −*x*+1, −*y*, −*z*; (xiii) −*x*+1, −*y*, *z*+1/2; (xiv) −*x*+*y*, −*x*+1, *z*; (xv) −*y*+1, *x*−*y*+1, *z*; (xvi) *y*, −*x*+*y*+1, −*z*; (xvii) −*x*+*y*+1, −*x*+1, *z*.

### Pentacalcium diyttrium heptasilicon oxynitride (3) Crystal data

**Table:**

Ca_5_Y_2_Si_7_O_16_N_4_	*D*_x_ = 3.359 Mg m^−^^3^
*M**_r_* = 886.89	Mo *K*α radiation, λ = 0.71073 Å
Hexagonal, *P*6_3_/*m*	Cell parameters from 7553 reflections
Hall symbol: -P 6c	θ = 3.1–27.4°
*a* = 10.0541 (2) Å	µ = 8.63 mm^−^^1^
*c* = 10.0168 (2) Å	*T* = 293 K
*V* = 876.89 (4) Å^3^	Platelet, colorless
*Z* = 2	0.05 × 0.03 × 0.02 mm
*F*(000) = 864	

### Pentacalcium diyttrium heptasilicon oxynitride (3) Data collection

**Table:**

Rigaku R-Axis RAPID II diffractometer	702 independent reflections
Radiation source: sealed x-ray tube	681 reflections with *I* > 2σ(*I*)
Graphite monochromator	*R*_int_ = 0.027
Detector resolution: 10 pixels mm^-1^	θ_max_ = 27.4°, θ_min_ = 3.1°
phi or ω oscillation scans	*h* = −13→13
Absorption correction: multi-scan (*ABSCOR*; Higashi, 2001)	*k* = −13→13
*T*_min_ = 0.820, *T*_max_ = 1.000	*l* = −12→12
8532 measured reflections	

### Pentacalcium diyttrium heptasilicon oxynitride (3) Refinement

**Table:**

Refinement on *F*^2^	0 constraints
Least-squares matrix: full	Primary atom site location: structure-invariant direct methods
*R*[*F*^2^ > 2σ(*F*^2^)] = 0.024	*w* = 1/[σ^2^(*F*_o_^2^) + (0.0366*P*)^2^ + 1.4061*P*] where *P* = (*F*_o_^2^ + 2*F*_c_^2^)/3
*wR*(*F*^2^) = 0.062	(Δ/σ)_max_ < 0.001
*S* = 1.09	Δρ_max_ = 0.50 e Å^−^^3^
702 reflections	Δρ_min_ = −0.86 e Å^−^^3^
63 parameters	Extinction correction: SHELXL-2014/7 (Sheldrick 2015b), Fc^*^=kFc[1+0.001xFc^2^λ^3^/sin(2θ)]^-1/4^
1 restraint	Extinction coefficient: 0.0135 (11)

### Pentacalcium diyttrium heptasilicon oxynitride (3) Special details

**Table:**

Geometry. All esds (except the esd in the dihedral angle between two l.s. planes) are estimated using the full covariance matrix. The cell esds are taken into account individually in the estimation of esds in distances, angles and torsion angles; correlations between esds in cell parameters are only used when they are defined by crystal symmetry. An approximate (isotropic) treatment of cell esds is used for estimating esds involving l.s. planes.

### Pentacalcium diyttrium heptasilicon oxynitride (3) Fractional atomic coordinates and isotropic or equivalent isotropic displacement parameters (Å^2^)

**Table:**

	*x*	*y*	*z*	*U*_iso_*/*U*_eq_	Occ. (<1)
Ca1	0	0	0	0.00811 (18)	0.2572 (14)
Y1	0	0	0	0.00811 (18)	0.7428 (14)
Ca2	0.41677 (5)	0.13055 (4)	0.03368 (4)	0.01268 (17)	0.7905 (2)
Y2	0.41677 (5)	0.13055 (4)	0.03368 (4)	0.01268 (17)	0.2095 (2)
Si1	0.11074 (9)	0.31954 (9)	0.25	0.0073 (2)	
Si2	0.55788 (9)	0.00978 (9)	0.25	0.0087 (2)	
Si3	0.3333	0.6667	0.21626 (19)	0.0077 (5)	0.5
O1	0.08563 (19)	0.22613 (19)	0.11149 (17)	0.0150 (4)	
O2	0.4430 (2)	0.35772 (19)	0.10933 (18)	0.0187 (4)	
O3	0.3963 (3)	0.0190 (3)	0.25	0.0197 (5)	
O4	0.3333	0.6667	0.0400 (6)	0.0452 (13)	0.5
N4	0.3333	0.6667	0.0400 (6)	0.0452 (13)	0.5
N5	0.2988 (3)	0.4832 (3)	0.25	0.0208 (7)	

### Pentacalcium diyttrium heptasilicon oxynitride (3) Atomic displacement parameters (Å^2^)

**Table:**

	*U*^11^	*U*^22^	*U*^33^	*U*^12^	*U*^13^	*U*^23^
Ca1	0.0084 (2)	0.0084 (2)	0.0076 (3)	0.00418 (10)	0	0
Y1	0.0084 (2)	0.0084 (2)	0.0076 (3)	0.00418 (10)	0	0
Ca2	0.0191 (2)	0.0123 (2)	0.0098 (2)	0.01028 (16)	−0.00029 (14)	−0.00046 (13)
Y2	0.0191 (2)	0.0123 (2)	0.0098 (2)	0.01028 (16)	−0.00029 (14)	−0.00046 (13)
Si1	0.0074 (4)	0.0071 (4)	0.0064 (4)	0.0029 (3)	0	0
Si2	0.0070 (4)	0.0089 (4)	0.0097 (4)	0.0035 (3)	0	0
Si3	0.0054 (5)	0.0054 (5)	0.0123 (14)	0.0027 (2)	0	0
O1	0.0173 (8)	0.0165 (8)	0.0097 (8)	0.0073 (7)	−0.0001 (6)	−0.0043 (7)
O2	0.0257 (9)	0.0186 (8)	0.0143 (8)	0.0130 (7)	−0.0049 (7)	−0.0055 (7)
O3	0.0185 (12)	0.0207 (12)	0.0206 (13)	0.0103 (10)	0	0
O4	0.0318 (14)	0.0318 (14)	0.072 (4)	0.0159 (7)	0	0
N4	0.0318 (14)	0.0318 (14)	0.072 (4)	0.0159 (7)	0	0
N5	0.0077 (12)	0.0102 (13)	0.043 (2)	0.0033 (10)	0	0

### Pentacalcium diyttrium heptasilicon oxynitride (3) Geometric parameters (Å, º)

**Table:**

Ca1—O1^i^	2.2804 (16)	Si2—Ca2^viii^	3.1472 (7)
Ca1—O1^ii^	2.2804 (16)	Si2—Ca2^xii^	3.2387 (5)
Ca1—O1	2.2804 (16)	Si2—Y2^xii^	3.2387 (5)
Ca1—O1^iii^	2.2804 (16)	Si2—Ca2^xiii^	3.2387 (5)
Ca1—O1^iv^	2.2804 (16)	Si2—Y2^xiii^	3.2387 (5)
Ca1—O1^v^	2.2804 (16)	Si3—Si3^viii^	0.676 (4)
Ca1—Ca2	3.7276 (4)	Si3—N5^xiv^	1.732 (3)
Ca1—Ca2^ii^	3.7276 (4)	Si3—N5^xv^	1.732 (3)
Ca1—Ca2^i^	3.7276 (4)	Si3—N5	1.732 (3)
Ca1—Y2^i^	3.7276 (4)	Si3—O4	1.765 (7)
Ca1—Y2^ii^	3.7276 (4)	Si3—Ca2^xvi^	3.4080 (15)
Ca2—O2	2.2930 (17)	Si3—Y2^xvi^	3.4080 (15)
Ca2—O3	2.4010 (12)	Si3—Ca2^i^	3.4080 (15)
Ca2—O2^iii^	2.4136 (18)	Si3—Y2^i^	3.4080 (15)
Ca2—N4^vi^	2.4274 (19)	Si3—Y2^vi^	3.4080 (15)
Ca2—O4^vi^	2.4274 (19)	Si3—Ca2^vi^	3.4080 (15)
Ca2—O1^iii^	2.4475 (17)	O1—Y2^i^	2.4475 (17)
Ca2—O2^vii^	2.6369 (18)	O1—Ca2^i^	2.4475 (17)
Ca2—O1^ii^	2.6475 (17)	O1—Ca2^iv^	2.6475 (17)
Ca2—N5^iii^	2.9071 (7)	O1—Y2^iv^	2.6475 (17)
Ca2—Si1^ii^	3.1432 (7)	O2—Si2^xvii^	1.6241 (18)
Ca2—Si2	3.1472 (7)	O2—Y2^i^	2.4136 (18)
Ca2—Si1^iii^	3.2277 (5)	O2—Ca2^i^	2.4136 (18)
Si1—O1^viii^	1.6227 (17)	O2—Ca2^xvii^	2.6370 (18)
Si1—O1	1.6228 (17)	O2—Y2^xvii^	2.6370 (18)
Si1—O3^iv^	1.673 (3)	O3—Si1^ii^	1.673 (3)
Si1—N5	1.781 (3)	O3—Ca2^viii^	2.4011 (12)
Si1—Ca2^iv^	3.1433 (7)	O3—Y2^viii^	2.4011 (12)
Si1—Y2^iv^	3.1433 (7)	O4—Y2^xvi^	2.4273 (19)
Si1—Ca2^ix^	3.1433 (7)	O4—Ca2^xvi^	2.4273 (19)
Si1—Y2^ix^	3.1433 (7)	O4—Ca2^vi^	2.4273 (19)
Si1—Ca2^x^	3.2277 (5)	O4—Y2^vi^	2.4273 (19)
Si1—Y2^x^	3.2277 (5)	O4—Y2^i^	2.4273 (19)
Si1—Y2^i^	3.2277 (5)	O4—Ca2^i^	2.4273 (19)
Si1—Ca2^i^	3.2277 (5)	N5—Si3^viii^	1.732 (3)
Si2—O2^xi^	1.6241 (18)	N5—Si2^xvii^	1.782 (3)
Si2—O2^vii^	1.6241 (18)	N5—Ca2^i^	2.9071 (7)
Si2—O3	1.672 (3)	N5—Y2^i^	2.9071 (7)
Si2—N5^vii^	1.782 (3)	N5—Y2^x^	2.9071 (7)
Si2—Y2^viii^	3.1472 (7)	N5—Ca2^x^	2.9071 (7)
			
O1^i^—Ca1—O1^ii^	180.00 (9)	O2^xi^—Si2—Y2^viii^	56.86 (7)
O1^i^—Ca1—O1	81.94 (6)	O2^vii^—Si2—Y2^viii^	130.39 (7)
O1^ii^—Ca1—O1	98.06 (6)	O3—Si2—Y2^viii^	48.78 (4)
O1^i^—Ca1—O1^iii^	98.06 (6)	N5^vii^—Si2—Y2^viii^	121.36 (6)
O1^ii^—Ca1—O1^iii^	81.94 (6)	O2^xi^—Si2—Ca2^viii^	56.86 (7)
O1—Ca1—O1^iii^	81.94 (6)	O2^vii^—Si2—Ca2^viii^	130.39 (7)
O1^i^—Ca1—O1^iv^	81.94 (6)	O3—Si2—Ca2^viii^	48.78 (4)
O1^ii^—Ca1—O1^iv^	98.06 (6)	N5^vii^—Si2—Ca2^viii^	121.36 (6)
O1—Ca1—O1^iv^	98.06 (6)	Y2^viii^—Si2—Ca2^viii^	0
O1^iii^—Ca1—O1^iv^	180.00 (12)	O2^xi^—Si2—Ca2	130.39 (7)
O1^i^—Ca1—O1^v^	98.06 (6)	O2^vii^—Si2—Ca2	56.86 (7)
O1^ii^—Ca1—O1^v^	81.94 (6)	O3—Si2—Ca2	48.78 (4)
O1—Ca1—O1^v^	180	N5^vii^—Si2—Ca2	121.36 (6)
O1^iii^—Ca1—O1^v^	98.06 (6)	Y2^viii^—Si2—Ca2	87
O1^iv^—Ca1—O1^v^	81.94 (6)	Ca2^viii^—Si2—Ca2	87.02 (2)
O1^i^—Ca1—Ca2	135.31 (4)	O2^xi^—Si2—Ca2^xii^	145.93 (8)
O1^ii^—Ca1—Ca2	44.69 (4)	O2^vii^—Si2—Ca2^xii^	46.05 (6)
O1—Ca1—Ca2	78.88 (4)	O3—Si2—Ca2^xii^	108.32 (4)
O1^iii^—Ca1—Ca2	39.57 (4)	N5^vii^—Si2—Ca2^xii^	63.15 (2)
O1^iv^—Ca1—Ca2	140.43 (4)	Y2^viii^—Si2—Ca2^xii^	157.1
O1^v^—Ca1—Ca2	101.12 (4)	Ca2^viii^—Si2—Ca2^xii^	157.07 (3)
O1^i^—Ca1—Ca2^ii^	101.12 (4)	Ca2—Si2—Ca2^xii^	73.312 (11)
O1^ii^—Ca1—Ca2^ii^	78.88 (4)	O2^xi^—Si2—Y2^xii^	145.93 (8)
O1—Ca1—Ca2^ii^	140.43 (4)	O2^vii^—Si2—Y2^xii^	46.05 (6)
O1^iii^—Ca1—Ca2^ii^	135.31 (4)	O3—Si2—Y2^xii^	108.32 (4)
O1^iv^—Ca1—Ca2^ii^	44.69 (4)	N5^vii^—Si2—Y2^xii^	63.15 (2)
O1^v^—Ca1—Ca2^ii^	39.57 (4)	Y2^viii^—Si2—Y2^xii^	157.07 (3)
Ca2—Ca1—Ca2^ii^	119.190 (2)	Ca2^viii^—Si2—Y2^xii^	157.07 (3)
O1^i^—Ca1—Ca2^i^	78.88 (4)	Ca2—Si2—Y2^xii^	73.3
O1^ii^—Ca1—Ca2^i^	101.12 (4)	Ca2^xii^—Si2—Y2^xii^	0.000 (15)
O1—Ca1—Ca2^i^	39.57 (4)	O2^xi^—Si2—Ca2^xiii^	46.05 (6)
O1^iii^—Ca1—Ca2^i^	44.69 (4)	O2^vii^—Si2—Ca2^xiii^	145.93 (8)
O1^iv^—Ca1—Ca2^i^	135.31 (4)	O3—Si2—Ca2^xiii^	108.32 (4)
O1^v^—Ca1—Ca2^i^	140.43 (4)	N5^vii^—Si2—Ca2^xiii^	63.15 (2)
Ca2—Ca1—Ca2^i^	60.810 (2)	Y2^viii^—Si2—Ca2^xiii^	73.3
Ca2^ii^—Ca1—Ca2^i^	180.000 (12)	Ca2^viii^—Si2—Ca2^xiii^	73.312 (11)
O1^i^—Ca1—Y2^i^	78.88 (4)	Ca2—Si2—Ca2^xiii^	157.07 (3)
O1^ii^—Ca1—Y2^i^	101.12 (4)	Ca2^xii^—Si2—Ca2^xiii^	122.65 (3)
O1—Ca1—Y2^i^	39.57 (4)	Y2^xii^—Si2—Ca2^xiii^	122.7
O1^iii^—Ca1—Y2^i^	44.69 (4)	O2^xi^—Si2—Y2^xiii^	46.05 (6)
O1^iv^—Ca1—Y2^i^	135.31 (4)	O2^vii^—Si2—Y2^xiii^	145.93 (8)
O1^v^—Ca1—Y2^i^	140.43 (4)	O3—Si2—Y2^xiii^	108.32 (4)
Ca2—Ca1—Y2^i^	60.8	N5^vii^—Si2—Y2^xiii^	63.15 (2)
Ca2^ii^—Ca1—Y2^i^	180.000 (12)	Y2^viii^—Si2—Y2^xiii^	73.312 (11)
Ca2^i^—Ca1—Y2^i^	0.000 (12)	Ca2^viii^—Si2—Y2^xiii^	73.312 (11)
O1^i^—Ca1—Y2^ii^	101.12 (4)	Ca2—Si2—Y2^xiii^	157.1
O1^ii^—Ca1—Y2^ii^	78.88 (4)	Ca2^xii^—Si2—Y2^xiii^	122.65 (3)
O1—Ca1—Y2^ii^	140.43 (4)	Y2^xii^—Si2—Y2^xiii^	122.65 (3)
O1^iii^—Ca1—Y2^ii^	135.31 (4)	Ca2^xiii^—Si2—Y2^xiii^	0.000 (16)
O1^iv^—Ca1—Y2^ii^	44.69 (4)	Si3^viii^—Si3—N5^xiv^	78.75 (7)
O1^v^—Ca1—Y2^ii^	39.57 (4)	Si3^viii^—Si3—N5^xv^	78.74 (7)
Ca2—Ca1—Y2^ii^	119.2	N5^xiv^—Si3—N5^xv^	116.29 (4)
Ca2^ii^—Ca1—Y2^ii^	0.000 (16)	Si3^viii^—Si3—N5	78.74 (6)
Ca2^i^—Ca1—Y2^ii^	180	N5^xiv^—Si3—N5	116.29 (4)
Y2^i^—Ca1—Y2^ii^	180	N5^xv^—Si3—N5	116.29 (4)
O2—Ca2—O3	96.16 (7)	Si3^viii^—Si3—O4	180
O2—Ca2—O2^iii^	161.05 (5)	N5^xiv^—Si3—O4	101.25 (6)
O3—Ca2—O2^iii^	100.98 (7)	N5^xv^—Si3—O4	101.25 (6)
O2—Ca2—N4^vi^	73.06 (8)	N5—Si3—O4	101.25 (6)
O3—Ca2—N4^vi^	118.54 (14)	Si3^viii^—Si3—Ca2^xvi^	137.28 (2)
O2^iii^—Ca2—N4^vi^	105.11 (11)	N5^xiv^—Si3—Ca2^xvi^	58.53 (4)
O2—Ca2—O4^vi^	73.06 (8)	N5^xv^—Si3—Ca2^xvi^	118.42 (8)
O3—Ca2—O4^vi^	118.54 (14)	N5—Si3—Ca2^xvi^	118.45 (8)
O2^iii^—Ca2—O4^vi^	105.11 (11)	O4—Si3—Ca2^xvi^	42.73 (2)
N4^vi^—Ca2—O4^vi^	0	Si3^viii^—Si3—Y2^xvi^	137.28 (2)
O2—Ca2—O1^iii^	81.84 (6)	N5^xiv^—Si3—Y2^xvi^	58.53 (4)
O3—Ca2—O1^iii^	132.03 (7)	N5^xv^—Si3—Y2^xvi^	118.42 (8)
O2^iii^—Ca2—O1^iii^	80.71 (6)	N5—Si3—Y2^xvi^	118.45 (8)
N4^vi^—Ca2—O1^iii^	106.72 (11)	O4—Si3—Y2^xvi^	42.73 (2)
O4^vi^—Ca2—O1^iii^	106.72 (11)	Ca2^xvi^—Si3—Y2^xvi^	0.00 (2)
O2—Ca2—O2^vii^	113.34 (8)	Si3^viii^—Si3—Ca2^i^	137.27 (2)
O3—Ca2—O2^vii^	62.62 (7)	N5^xiv^—Si3—Ca2^i^	118.42 (8)
O2^iii^—Ca2—O2^vii^	82.05 (6)	N5^xv^—Si3—Ca2^i^	118.45 (8)
N4^vi^—Ca2—O2^vii^	67.30 (8)	N5—Si3—Ca2^i^	58.53 (4)
O4^vi^—Ca2—O2^vii^	67.30 (8)	O4—Si3—Ca2^i^	42.73 (2)
O1^iii^—Ca2—O2^vii^	159.44 (6)	Ca2^xvi^—Si3—Ca2^i^	71.97 (4)
O2—Ca2—O1^ii^	105.75 (6)	Y2^xvi^—Si3—Ca2^i^	72
O3—Ca2—O1^ii^	62.69 (7)	Si3^viii^—Si3—Y2^i^	137.27 (2)
O2^iii^—Ca2—O1^ii^	75.59 (5)	N5^xiv^—Si3—Y2^i^	118.42 (8)
N4^vi^—Ca2—O1^ii^	178.26 (6)	N5^xv^—Si3—Y2^i^	118.45 (8)
O4^vi^—Ca2—O1^ii^	178.26 (6)	N5—Si3—Y2^i^	58.53 (4)
O1^iii^—Ca2—O1^ii^	71.75 (7)	O4—Si3—Y2^i^	42.73 (2)
O2^vii^—Ca2—O1^ii^	114.42 (5)	Ca2^xvi^—Si3—Y2^i^	71.97 (4)
O2—Ca2—N5^iii^	103.67 (7)	Y2^xvi^—Si3—Y2^i^	71.97 (4)
O3—Ca2—N5^iii^	157.98 (8)	Ca2^i^—Si3—Y2^i^	0.00 (2)
O2^iii^—Ca2—N5^iii^	61.16 (7)	Si3^viii^—Si3—Y2^vi^	137.27 (2)
N4^vi^—Ca2—N5^iii^	60.10 (15)	N5^xiv^—Si3—Y2^vi^	118.45 (8)
O4^vi^—Ca2—N5^iii^	60.10 (15)	N5^xv^—Si3—Y2^vi^	58.53 (4)
O1^iii^—Ca2—N5^iii^	61.53 (7)	N5—Si3—Y2^vi^	118.42 (8)
O2^vii^—Ca2—N5^iii^	100.12 (7)	O4—Si3—Y2^vi^	42.73 (2)
O1^ii^—Ca2—N5^iii^	119.31 (7)	Ca2^xvi^—Si3—Y2^vi^	71.97 (4)
O2—Ca2—Si1^ii^	103.99 (5)	Y2^xvi^—Si3—Y2^vi^	71.97 (4)
O3—Ca2—Si1^ii^	31.66 (6)	Ca2^i^—Si3—Y2^vi^	71.97 (4)
O2^iii^—Ca2—Si1^ii^	86.88 (4)	Y2^i^—Si3—Y2^vi^	71.97 (4)
N4^vi^—Ca2—Si1^ii^	150.19 (13)	Si3^viii^—Si3—Ca2^vi^	137.27 (2)
O4^vi^—Ca2—Si1^ii^	150.19 (13)	N5^xiv^—Si3—Ca2^vi^	118.45 (8)
O1^iii^—Ca2—Si1^ii^	102.05 (4)	N5^xv^—Si3—Ca2^vi^	58.53 (4)
O2^vii^—Ca2—Si1^ii^	88.07 (4)	N5—Si3—Ca2^vi^	118.42 (8)
O1^ii^—Ca2—Si1^ii^	31.07 (4)	O4—Si3—Ca2^vi^	42.73 (2)
N5^iii^—Ca2—Si1^ii^	145.05 (6)	Ca2^xvi^—Si3—Ca2^vi^	71.97 (4)
O2—Ca2—Si2	107.89 (5)	Y2^xvi^—Si3—Ca2^vi^	72
O3—Ca2—Si2	31.59 (6)	Ca2^i^—Si3—Ca2^vi^	71.97 (4)
O2^iii^—Ca2—Si2	91.00 (5)	Y2^i^—Si3—Ca2^vi^	72
N4^vi^—Ca2—Si2	93.24 (10)	Y2^vi^—Si3—Ca2^vi^	0
O4^vi^—Ca2—Si2	93.24 (10)	Si1—O1—Ca1	150.18 (10)
O1^iii^—Ca2—Si2	159.75 (4)	Si1—O1—Y2^i^	103.06 (8)
O2^vii^—Ca2—Si2	31.04 (4)	Ca1—O1—Y2^i^	104.02 (6)
O1^ii^—Ca2—Si2	88.33 (4)	Si1—O1—Ca2^i^	103.06 (8)
N5^iii^—Ca2—Si2	129.71 (5)	Ca1—O1—Ca2^i^	104.02 (6)
Si1^ii^—Ca2—Si2	58.79 (2)	Y2^i^—O1—Ca2^i^	0
O2—Ca2—Si1^iii^	86.92 (5)	Si1—O1—Ca2^iv^	91.58 (7)
O3—Ca2—Si1^iii^	160.52 (6)	Ca1—O1—Ca2^iv^	98.02 (6)
O2^iii^—Ca2—Si1^iii^	74.24 (5)	Y2^i^—O1—Ca2^iv^	95.5
N4^vi^—Ca2—Si1^iii^	80.79 (13)	Ca2^i^—O1—Ca2^iv^	95.48 (6)
O4^vi^—Ca2—Si1^iii^	80.79 (13)	Si1—O1—Y2^iv^	91.58 (7)
O1^iii^—Ca2—Si1^iii^	29.32 (4)	Ca1—O1—Y2^iv^	98.02 (6)
O2^vii^—Ca2—Si1^iii^	133.34 (4)	Y2^i^—O1—Y2^iv^	95.48 (6)
O1^ii^—Ca2—Si1^iii^	97.93 (4)	Ca2^i^—O1—Y2^iv^	95.48 (6)
N5^iii^—Ca2—Si1^iii^	33.23 (5)	Ca2^iv^—O1—Y2^iv^	0.000 (18)
Si1^ii^—Ca2—Si1^iii^	129.00 (3)	Si2^xvii^—O2—Ca2	139.12 (11)
Si2—Ca2—Si1^iii^	161.82 (2)	Si2^xvii^—O2—Y2^i^	104.98 (8)
O1^viii^—Si1—O1	117.51 (13)	Ca2—O2—Y2^i^	106.5
O1^viii^—Si1—O3^iv^	106.16 (8)	Si2^xvii^—O2—Ca2^i^	104.98 (8)
O1—Si1—O3^iv^	106.16 (8)	Ca2—O2—Ca2^i^	106.55 (7)
O1^viii^—Si1—N5	108.71 (8)	Y2^i^—O2—Ca2^i^	0
O1—Si1—N5	108.71 (8)	Si2^xvii^—O2—Ca2^xvii^	92.10 (8)
O3^iv^—Si1—N5	109.35 (13)	Ca2—O2—Ca2^xvii^	108.46 (7)
O1^viii^—Si1—Ca2^iv^	129.73 (7)	Y2^i^—O2—Ca2^xvii^	98
O1—Si1—Ca2^iv^	57.35 (6)	Ca2^i^—O2—Ca2^xvii^	97.95 (6)
O3^iv^—Si1—Ca2^iv^	48.90 (4)	Si2^xvii^—O2—Y2^xvii^	92.10 (8)
N5—Si1—Ca2^iv^	120.32 (6)	Ca2—O2—Y2^xvii^	108.5
O1^viii^—Si1—Y2^iv^	129.73 (7)	Y2^i^—O2—Y2^xvii^	97.95 (6)
O1—Si1—Y2^iv^	57.35 (6)	Ca2^i^—O2—Y2^xvii^	97.95 (6)
O3^iv^—Si1—Y2^iv^	48.90 (4)	Ca2^xvii^—O2—Y2^xvii^	0.00 (2)
N5—Si1—Y2^iv^	120.32 (6)	Si2—O3—Si1^ii^	134.77 (16)
Ca2^iv^—Si1—Y2^iv^	0.00 (2)	Si2—O3—Ca2	99.63 (7)
O1^viii^—Si1—Ca2^ix^	57.35 (6)	Si1^ii^—O3—Ca2	99.44 (7)
O1—Si1—Ca2^ix^	129.73 (7)	Si2—O3—Ca2^viii^	99.63 (7)
O3^iv^—Si1—Ca2^ix^	48.90 (4)	Si1^ii^—O3—Ca2^viii^	99.44 (7)
N5—Si1—Ca2^ix^	120.32 (6)	Ca2—O3—Ca2^viii^	128.97 (11)
Ca2^iv^—Si1—Ca2^ix^	87.16 (2)	Si2—O3—Y2^viii^	99.63 (7)
Y2^iv^—Si1—Ca2^ix^	87.2	Si1^ii^—O3—Y2^viii^	99.44 (7)
O1^viii^—Si1—Y2^ix^	57.35 (6)	Ca2—O3—Y2^viii^	129
O1—Si1—Y2^ix^	129.73 (7)	Ca2^viii^—O3—Y2^viii^	0.00 (4)
O3^iv^—Si1—Y2^ix^	48.90 (4)	Si3—O4—Y2^xvi^	107.71 (14)
N5—Si1—Y2^ix^	120.32 (6)	Si3—O4—Ca2^xvi^	107.71 (14)
Ca2^iv^—Si1—Y2^ix^	87.16 (2)	Y2^xvi^—O4—Ca2^xvi^	0
Y2^iv^—Si1—Y2^ix^	87.16 (2)	Si3—O4—Ca2^vi^	107.71 (14)
Ca2^ix^—Si1—Y2^ix^	0.00 (2)	Y2^xvi^—O4—Ca2^vi^	111.2
O1^viii^—Si1—Ca2^x^	47.62 (6)	Ca2^xvi^—O4—Ca2^vi^	111.18 (13)
O1—Si1—Ca2^x^	146.23 (7)	Si3—O4—Y2^vi^	107.71 (14)
O3^iv^—Si1—Ca2^x^	107.33 (4)	Y2^xvi^—O4—Y2^vi^	111.18 (13)
N5—Si1—Ca2^x^	63.45 (2)	Ca2^xvi^—O4—Y2^vi^	111.18 (13)
Ca2^iv^—Si1—Ca2^x^	156.21 (3)	Ca2^vi^—O4—Y2^vi^	0.00 (3)
Y2^iv^—Si1—Ca2^x^	156.2	Si3—O4—Y2^i^	107.71 (14)
Ca2^ix^—Si1—Ca2^x^	72.618 (6)	Y2^xvi^—O4—Y2^i^	111.18 (13)
Y2^ix^—Si1—Ca2^x^	72.6	Ca2^xvi^—O4—Y2^i^	111.18 (13)
O1^viii^—Si1—Y2^x^	47.62 (6)	Ca2^vi^—O4—Y2^i^	111.18 (13)
O1—Si1—Y2^x^	146.23 (7)	Y2^vi^—O4—Y2^i^	111.18 (13)
O3^iv^—Si1—Y2^x^	107.33 (4)	Si3—O4—Ca2^i^	107.71 (14)
N5—Si1—Y2^x^	63.45 (2)	Y2^xvi^—O4—Ca2^i^	111.2
Ca2^iv^—Si1—Y2^x^	156.21 (3)	Ca2^xvi^—O4—Ca2^i^	111.18 (13)
Y2^iv^—Si1—Y2^x^	156.21 (3)	Ca2^vi^—O4—Ca2^i^	111.18 (13)
Ca2^ix^—Si1—Y2^x^	72.618 (6)	Y2^vi^—O4—Ca2^i^	111.2
Y2^ix^—Si1—Y2^x^	72.618 (6)	Y2^i^—O4—Ca2^i^	0
Ca2^x^—Si1—Y2^x^	0.00 (3)	Si3^viii^—N5—Si3	22.51 (13)
O1^viii^—Si1—Y2^i^	146.23 (7)	Si3^viii^—N5—Si1	122.61 (15)
O1—Si1—Y2^i^	47.62 (6)	Si3—N5—Si1	122.61 (15)
O3^iv^—Si1—Y2^i^	107.33 (4)	Si3^viii^—N5—Si2^xvii^	120.70 (15)
N5—Si1—Y2^i^	63.45 (2)	Si3—N5—Si2^xvii^	120.70 (15)
Ca2^iv^—Si1—Y2^i^	72.618 (6)	Si1—N5—Si2^xvii^	115.29 (16)
Y2^iv^—Si1—Y2^i^	72.618 (6)	Si3^viii^—N5—Ca2^i^	113.45 (10)
Ca2^ix^—Si1—Y2^i^	156.21 (3)	Si3—N5—Ca2^i^	90.94 (7)
Y2^ix^—Si1—Y2^i^	156.21 (3)	Si1—N5—Ca2^i^	83.32 (6)
Ca2^x^—Si1—Y2^i^	123.37 (3)	Si2^xvii^—N5—Ca2^i^	83.70 (6)
Y2^x^—Si1—Y2^i^	123.37 (3)	Si3^viii^—N5—Y2^i^	113.45 (10)
O1^viii^—Si1—Ca2^i^	146.23 (7)	Si3—N5—Y2^i^	90.94 (7)
O1—Si1—Ca2^i^	47.62 (6)	Si1—N5—Y2^i^	83.32 (6)
O3^iv^—Si1—Ca2^i^	107.33 (4)	Si2^xvii^—N5—Y2^i^	83.70 (6)
N5—Si1—Ca2^i^	63.45 (2)	Ca2^i^—N5—Y2^i^	0.00 (3)
Ca2^iv^—Si1—Ca2^i^	72.618 (6)	Si3^viii^—N5—Y2^x^	90.94 (7)
Y2^iv^—Si1—Ca2^i^	72.6	Si3—N5—Y2^x^	113.45 (10)
Ca2^ix^—Si1—Ca2^i^	156.21 (3)	Si1—N5—Y2^x^	83.32 (6)
Y2^ix^—Si1—Ca2^i^	156.2	Si2^xvii^—N5—Y2^x^	83.70 (6)
Ca2^x^—Si1—Ca2^i^	123.37 (3)	Ca2^i^—N5—Y2^x^	155.61 (11)
Y2^x^—Si1—Ca2^i^	123.4	Y2^i^—N5—Y2^x^	155.61 (11)
Y2^i^—Si1—Ca2^i^	0	Si3^viii^—N5—Ca2^x^	90.94 (7)
O2^xi^—Si2—O2^vii^	120.36 (13)	Si3—N5—Ca2^x^	113.45 (10)
O2^xi^—Si2—O3	105.60 (8)	Si1—N5—Ca2^x^	83.32 (6)
O2^vii^—Si2—O3	105.60 (8)	Si2^xvii^—N5—Ca2^x^	83.70 (6)
O2^xi^—Si2—N5^vii^	107.03 (8)	Ca2^i^—N5—Ca2^x^	155.61 (11)
O2^vii^—Si2—N5^vii^	107.03 (8)	Y2^i^—N5—Ca2^x^	155.6
O3—Si2—N5^vii^	111.18 (13)	Y2^x^—N5—Ca2^x^	0

Symmetry codes: (i) *x*−*y*, *x*, −*z*; (ii) −*x*+*y*, −*x*, *z*; (iii) *y*, −*x*+*y*, −*z*; (iv) −*y*, *x*−*y*, *z*; (v) −*x*, −*y*, −*z*; (vi) −*x*+1, −*y*+1, −*z*; (vii) −*y*+1, *x*−*y*, *z*; (viii) *x*, *y*, −*z*+1/2; (ix) −*y*, *x*−*y*, −*z*+1/2; (x) *x*−*y*, *x*, *z*+1/2; (xi) −*y*+1, *x*−*y*, −*z*+1/2; (xii) −*x*+1, −*y*, −*z*; (xiii) −*x*+1, −*y*, *z*+1/2; (xiv) −*x*+*y*, −*x*+1, *z*; (xv) −*y*+1, *x*−*y*+1, *z*; (xvi) *y*, −*x*+*y*+1, −*z*; (xvii) −*x*+*y*+1, −*x*+1, *z*.
